# Acute and chronic central nervous system oxidative stress/toxicity during hyperbaric oxygen treatment of subacute and chronic neurological conditions

**DOI:** 10.3389/fneur.2024.1341562

**Published:** 2024-03-04

**Authors:** Paul G. Harch, Stacey Rhodes

**Affiliations:** ^1^Family Physician Center, Marrero, LA, United States; ^2^Department of Emergency Medicine, University Medical Center, New Orleans, LA, United States; ^3^Section of Emergency Medicine, Department of Medicine, Louisiana State University, New Orleans, LA, United States

**Keywords:** oxidative stress, oxygen toxicity, hyperbaric oxygen, treatment, chronic neurologic conditions

## Abstract

**Introduction:**

Oxygen toxicity has been defined as acute central nervous system (CNS), acute pulmonary, and chronic pulmonary oxygen toxicity. This study identifies acute and chronic CNS oxygen toxicity under 2.0 atmospheres absolute (ATA) pressure of oxygen. Methods: The authors’ medical records from September 29, 1989 to January 20, 2023 and correspondence to the authors (9/1994 to 1/20.2023) from patients with signs and/or symptoms historically identified as acute CNS oxygen toxicity and those with neurological deterioration receiving hyperbaric oxygen for neurological conditions were reviewed. Acute cases were those occurring with ≤5 HBOTs and chronic cases >5 HBOTs. Chronic cases were separated into those at 1.5 ATA, > 1.5 ATA, or < 1.5 ATA oxygen. Cumulative dose of oxygen in atmosphere-hours (AHs) was calculated at symptom onset.

**Results:**

Seven acute cases, average 4.0 ± 2.7 AHs, and 52 chronic cases were identified: 31 at 1.5 ATA (average 116 ± 106 AHs), 12 at >1.5 ATA (103 ± 74 AHs), and 9 at <1.5 ATA (114 ± 116 AHs). Second episodes occurred at 81 ± 55, 67 ± 49, and 22 ± 17 AHs, and three or more episodes at 25 ± 18, 83 ± 7.5, and 5.4 ± 6.0 AHs, respectively. Most cases were reversible. There was no difference between adults and children (*p* = 0.72). Acute intervention in cases (<3 months) was more sensitive than delayed intervention (21.1 ± 8.8 vs. 123 ± 102 AHs, *p* = 0.035). Outside sources reported one acute and two chronic exposure deaths and one patient institutionalized due to chronic oxygen toxicity. A withdrawal syndrome was also identified.

**Conclusion:**

Hyperbaric oxygen therapy-generated acute and chronic cases of CNS oxygen toxicity in chronic neurological conditions were identified at <2.0 ATA. Chronic CNS oxygen toxicity is idiosyncratic, unpredictable, and occurred at an average threshold of 103–116 AHs with wide variability. There was no difference between adults and children, but subacute cases were more sensitive than chronic intervention cases. When identified early it was reversible and an important aid in proper dosing of HBOT. If ignored permanent morbidity and mortality resulted with continued HBOT.

## 1 Introduction

Oxidative stress/toxicity (OT) in humans is a function of partial pressure of oxygen and time of exposure (1) and is thought to be caused by damage from oxygen free radicals (2, 3). It has been shown to occur in nearly all tissues and organs (4). The most sensitive target organs are the lungs and central nervous system (1) (CNS: brain and spinal cord) and the manifestations are usually separated into acute toxicity from continuous exposure and chronic toxicity from repetitive exposures. Acute CNS OT from continuous exposure to oxygen was first described in animals in 1878 with hyperbaric exposures and is known as the Paul Bert Effect (5). Acute pulmonary OT from continuous exposure to oxygen was first described in 1899 with normobaric and hyperbaric exposures and is known as the Lorrain Smith Effect (6). Chronic or cumulative pulmonary OT from repetitive exposure at hyperbaric pressures has been described and led to the development of the unit pulmonary toxic dose (UPTD) metric in divers (7–9). Chronic or cumulative CNS OT from repetitive hyperbaric exposures has been implied and replicated in animals (10–12), but dismissed/attributed to other causes in humans (13–16). It has only been described from continuous exposures of hours to days and consisted of paresthesias and numbness in fingers and toes, headache, dizziness, nausea, and reduction in aerobic capacity (17).

The research and clinical experience on CNS OT under hyperbaric conditions has been conducted and reported at ≥2.0 ATA oxygen on neurologically normal subjects (18–20), or emergency indication patients (13–15). These studies report the extreme manifestation of acute CNS OT, grand mal seizures. Due to the low incidence of seizures at 2.0 ATA and the traditionally greater clinical levels of 2.36–2.4 ATA (13–15) CNS OT under hyperbaric conditions was considered non-existent or impossible to elicit below 2.0 ATA (21–26) or more recently 1.9 ATA (27). Even when obvious CNS symptoms developed from continuous exposure at 2.0 ATA “…the cause of the symptoms is not known” (22) or the symptoms were characterized as a “somatic” constitutional form of OT (28), omitting the descriptor “chronic” to avoid confusion with chronic pulmonary OT (29). The rectangular hyperbola pressure- continuous time exposure graph of acute CNS OT supports this belief (30) and has been reinforced by a lack of identification and reporting of cumulative CNS OT with repetitive HBOT under 1.9–2.0 ATA. This may be due to the reporting of CNS OT from the common clinical use of HBOT at pressures of 2.0 ATA or greater (31–39), but more likely is due to the lack of application of HBOT to chronic neurological conditions prior to Neubauer (40).

Based on Neubauer’s work (40), the absence of reported CNS OT, and belief of non-existence of CNS OT below 1.9–2.0 ATA (21–27), Harch, Van Meter, and Gottlieb investigated the treatment of chronic neurological disorders with 40 treatment blocks of HBOT at 1.5 ATA/90 min daily from 1994 to 1999 (41). Cases with unanticipated untoward symptoms emerged in a group of carbon monoxide- poisoned patients with neuro-cognitive residual symptoms treated 6 months post carbon monoxide exposure (Reported at the Undersea and Hyperbaric Medical Society Gulf Coast Chapter Meeting 4/1995, New Orleans). Additional cases from multiple sources at exposures >1.5 ATA oxygen, prompted a cautionary statement to a worldwide parent support group for disabled children, the MUMS Network, in 1998 (reprinted 2022) (42) and a presentation of cumulative cases at the 2nd International Symposium on Hyperbaric Oxygenation and the Brain Injured Child in 1999 (43). In the subsequent 24 years the increasing neurological off-label use of HBOT by untrained medical and non-medical personnel, home use of hyperbaric therapy, continued contact of the primary author by patients with negative outcomes from HBOT for neurological disorders, and reports of permanent injury and death indicated a need for more widespread understanding of safe dosing limits of HBOT for chronic neurological disorders. This retrospective study addresses this need. Herein we report acute CNS OT within 5 HBOTs and chronic CNS OT with repetitive HBOT exposures, both at less than 2.0 ATA oxygen.

## 2 Materials and methods

Medical Records Archive and Search: The primary author maintained archives of all medical records of his hyperbaric patients from the time of the first application of hyperbaric oxygen therapy to divers with chronic decompression illness (September 29, 1989) and of all communications since 9/1994 from outside sources (physicians, hyperbaric medical facilities, families, and all individuals who sought consultation regarding hyperbaric oxygen therapy, particularly for complications of hyperbaric treatment) by phone, email, letter, or from a subsequent formal medical history and physical exam. The entire communication archive was searched from 9/1994 through Institutional Review Board approval of January 20, 2023 and a partial search of the medical records, mostly from the author’s memory and separately archived cases with complications, was conducted from September 29, 1989 to January 20, 2023.

Type of Records Selected: Records were selected that featured signs and symptoms (SS) of oxygen toxicity described by Donald (19, 20) that occurred during treatment with HBOT for chronic neurological conditions. These SS were change in level of consciousness (loss of consciousness, drowsiness, sleepiness, amnesia, confusion, loss of judgment), neuromuscular signs (clonic spasms of the legs, “blubbering of the lips,” muscle fasciculations, clumsiness, fibrillation of lips, lip twitching, twitching of cheek and nose or any muscle), salivation, behavioral SS (changes of behavior, fidgeting, disinterest), mood changes (depression, euphoria, apprehension, acute terror), autonomic SS (facial perspiration, diaphoresis, facial pallor, bradycardia, palpitations, sensation of arterial pulsation throughout the body, syncope); respiratory SS: panting, grunting, hiccoughs, inspiratory predominance, spasmotic respiration, choking sensation, epigastric tensions, epigastric aura, constriction or same in precordium; alteration of senses: paresthesias, visual symptoms (flashes of light, haloes, loss of acuity, lateral movement of images, decrease of intensity, constriction of visual field, hallucinations, micropsia), acoustic symptoms (music, bell ringing, knocking), hallucinations (unpleasant olfactory or gustatory sensations); malaise, spasmotic vomiting, vertigo, and SS in the acronym VENTIDS (visual, ear, nausea, twitching, irritability, dizziness, seizure). Synonyms were allowed, e.g., lethargy, lassitude, fatigue, and brady-kinesia for drowsiness or sleepiness.

Data Extraction, Analysis, and Statistics: Data from enrolled cases were extracted and a clinical vignette composed for each case. Data included age, sex, diagnosis, time from injury to HBOT, the HBOT schedule/dose, number of HBOTs at time of OT symptom/sign onset, the SS of OT, and cumulative oxygen dose at the onset of SS. Cumulative dose was calculated in atmosphere-hours (AHs) with the following equation:


$$\begin{array}{l} AHs=\mathrm{Depth}\;\left(ATA\right)\times \mathrm{Time of oxygen exposure}\;\left(\mathrm{hrs}.\right)\\ \;\times \mathrm{Number of HBOTs}\text{.} \end{array}$$

Cases were identified as the authors’ or from outside sources and were sorted by their historical chronological occurrence. The first cases were observed at 1.5 ATA during the 1994 clinical trial (*vide supra*). They were segregated from cases treated at > or < 1.5 ATA oxygen. Chamber type was designated as hardshell (steel and acrylic, typically, with a pressure limit of 3.0 ATA or greater) or softshell (synthetic soft material composition and pressure limits of 1.3 ATA). Functional brain imaging was included when available. SPECT scanner specifications, radiopharmaceutical, acquisition, and processing are described in Supplementary File S1 (44, 45). This study was approved as LSU IRB #4574. GraphPad^1^ was used to perform unpaired t tests on group comparisons. Means were calculated using Calculator.net.^2^

## 3 Results

Eighty-two cases were enrolled. Twenty-two cases were excluded due to equivocal signs and symptoms, critical missing data, or attribution to alternative explanations of SS, leaving 60 cases for the final cohort. These comprised 27 cases from a previous IRB-approved study and 33 cases since 7/2001. The study was closed at 60 cases, arbitrarily judged by us to be sufficiently representative of chronic CNS OT.

Abbreviated histories of all cases are in Supplementary File S2. Case reports from outside sources used the mother, family member, or immediate caregiver’s quotation of signs and symptoms. During the review acute CNS OT cases were identified from outside clinics. Five HBOTs were chosen as the cutoff for acuity (32–35). Included in the chronic cases were patients who demonstrated initial improvement in neurological and cognitive function with HBOT then reversed these improvements with continued HBOT, re-expressing their neuro-cognitive deficits, and finally regained their HBOT- induced improvements after discontinuance of HBOT. This pattern strongly suggested the cumulative oxidative stress with HBOT and recovery from same on discontinuance of HBOT described by Bean and Siegfried (46). The pattern was also indicative of typical drug overdosing and recovery upon discontinuance of the drug. As a result, this pattern was identified as a CNS OT manifestation.

Data from the Supplementary File S2 case reports are presented in Supplementary Tables S1–S5 and in Supplementary Table S1 (acute oxygen toxicity), Supplementary Table S2 (chronic CNS oxygen toxicity at 1.5 ATA oxygen), Supplementary Table S3 (chronic CNS oxygen toxicity at >1.5 ATA oxygen), Supplementary Table S4 (chronic CNS oxygen toxicity at <1.5 ATA oxygen), and Supplementary Table S5 (single case of withdrawal syndrome). Data from Supplementary Tables S1-S4 are condensed in Table 1.

**Table 1:** Acute and chronic CNS OT essential data from Supplementary Tables S1-S4.

	Oxygen pressure at occurrence of OT (ATA)	Number of cases; gender (% M, F)	Mean age ± (SD)	Mean AHs ± (SD) 1st OT	Mean AHs ± (SD) 2nd OT	Mean AHs ± (SD) ≥ 3rd OT
Acute O_2_ toxicity	1.5 1.5 ≤ 2.4 <1.5	1; F 5; M (100%) 1; M	32 24.6 (23.4)* 95	3 4.7 (3.0) 1.7**	– – 0.36	– – –
Chronic O_2_ toxicity	1.5 1.5 < 2.8 <1.5	31; M (61%) 12; M (75%) 9; M (44%)	30.3 (22.6)* 19.2 (16.3) 30.8 (25.5)	116 (106) 103 (74) 114 (116)	81 (55)^&^ 67 (49) 22 (17)	25 (18) 83 (7.5) 5.4 (6.0)

AH, Atmosphere Hours of oxygen exposure; OT, Oxygen Toxicity; SD, Standard Deviation. *Age was estimated on two subjects where exact age was unknown. **Patient with cumulative 4052 AHs upto few days before acute stroke then oxygen toxicity after 2nd subsequent HBOT. ^&^Excludes one outlier.

Supplementary Table S1 contains 7 cases of acute CNS OT, most of which (5 cases) occurred at ≤2.0 ATA. They were 2–95 y.o., 6 males, 1 female, with 7 different neurological diagnoses (two with seizure disorders). Five of the cases were in the chronic disease phase (greater than 6 months from time of injury, insult, infection, diagnosis). The single mortality was a 95 y.o. male with 3,000 previous HBOTs for a stroke who was treated a few hours after an acute stroke, experienced CNS OT on the second treatment, a grand mal seizure on the 3rd HBOT (1.1 ATA oxygen), 15 subsequent grand mal seizures in the next 24 h, and died after the 15th seizure.

**Figure 1:**
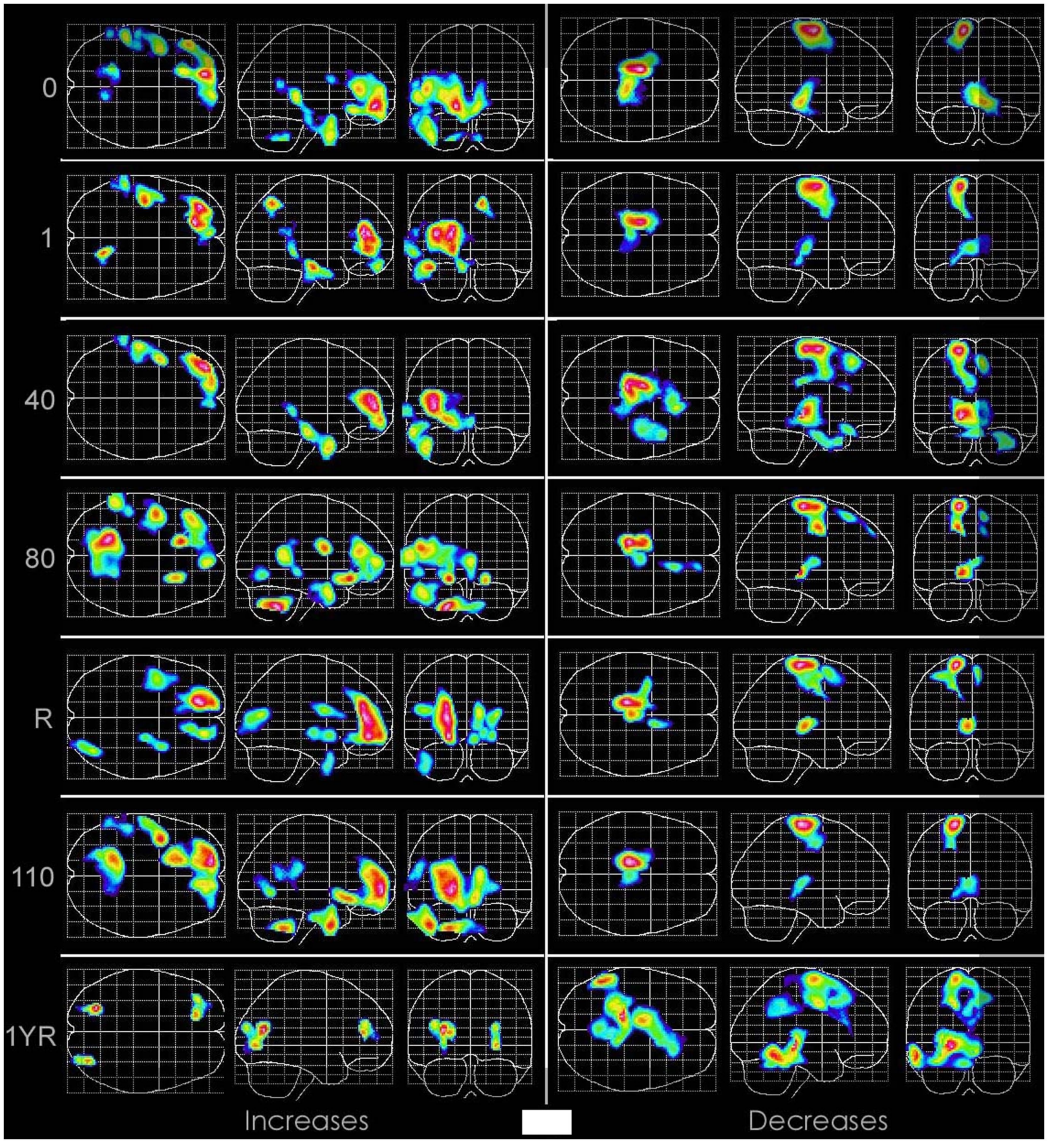
Sequential SPECT brain imaging of Subject #16 before the first HBOT (Row 0) then after 1, 40, 80 HBOTs, a 6-month break (Row R), 110 HBOTs, and 1 year post 110th HBOT (1 YR). Images are statistical parametric maps of selective coregistered slices. Color scheme: black indicates rCBF is within reference normal range. Increasing Z-scores (blood flow) are blue, green, orange, red, white on the left 3 columns and decreasing Z-scores with the same color scheme on the right 3 columns. SPECT at time 0 shows multiple areas of abnormally increased brain blood flow (top row left 3 images) and abnormally decreased brain blood flow (top row right 3 images) compared to normal and the other 6 patients in the study. Note intensification and broadening of Row R increased right frontal blood flow (6 months after 80th HBOT) that shows persistence and increases in the right frontal, posterior fronto-parietal and parietal, opposite anterior frontal, right temporal, and cerebellar areas after removal from the study for behavioral deterioration (Row 110), nearly all of which recede 1 year post study (1YR) to less than pre-HBOT levels (Row 0). Simultaneously, at 1YR post HBOT the right 3 panels show broadening of the adjacent areas with significant decreases in blood flow compared to the pre-HBOT level. Reproduced with permission Best Publishing Company (43).

Supplementary Table S2 lists 31 cases of chronic CNS OT after >5 HBOTs and at 1.5 ATA oxygen that include the first two unrecognized cases treated by the authors in 1990 and 1992 (47). The patients were 18 months to 60 y.o., 19 M, 12 F, 4 weeks to 10 years post injury/insult (29 ≥ 6 months since injury/insult), with 13 different neurological diagnoses. There were two severe complications from outside sources occurring in home hardshell chambers (one death and one institutionalization for severe behavioral deterioration). One case’s OT (#16) was serendipitously captured on SPECT brain blood flow imaging (Figure 1) in an IRB-approved study of HBOT in chronic severe TBI. There were nine cases with second episodes of CNS OT and six cases with three or more episodes.

Supplementary Table S3 contains 12 chronic CNS OT cases occurring at >1.5 ATA oxygen pressure. There were 9 males, 3 females, ages 2–58 y.o., 8d to 12 years post injury/insult, with 9 different diagnoses. There was one death from an outside source in a home hardshell chamber. The OT in six of 12 cases occurred after an escalation of oxygen pressure or on intensive twice/day or 6–7d/week schedules. Seven of the 12 cases occurred at 1.75 ATA, one at 1.55 ATA at 6,900 feet altitude, two at 2.0 ATA, one at 2.4 ATA, and one at 2.8 ATA. Second episodes of OT were documented in 5 cases and three or more episodes of CNS OT in one case.

Supplementary Table S4 contains nine chronic CNS OT cases occurring at <1.5 ATA oxygen pressure. There were 4 males, 5 females, ages 2.5–83 y.o., 2.5 months to 11 years after injury/insult with seven different neurological primary diagnoses. Four cases of OT occurred in portable chambers, two of whom occurred after prolonged chamber treatments (2–3 h each) or a prolonged course of treatment (135 HBOTs), and a fifth with post-surgical normobaric oxygen after long-term HBOT in a portable chamber. One was a subacutely drowned child who could not tolerate 1.15 ATA/45 min of compressed air. Overall, 4/9 had at least one episode while receiving compressed air at ≤1.5 ATA. Second episodes of CNS OT were documented in five cases and three or more episodes occurred in 3 patients.

Supplementary Table S5 lists a single case of apparent drug withdrawal that occurred after 130 HBOTs, the last 70 of which were consecutive, 7d/week, twice/day treatments. In the subsequent 2 weeks she experienced regression of her gains followed by a marked worsening of her pre-HBOT dystonia, involuntary total body movements, and teeth-grinding.

The critical data from Supplementary Tables S1-S4 are condensed in Table 1. The acute and chronic cases occurred over similar wide age ranges. Acute cases experienced CNS OT at <5 AHs while the chronic cases occurred regardless of pressure over a wide range of cumulative oxygen exposures with mean of 103–116 AHs. Second and third episodes of chronic CNS OT occurred with lesser additional cumulative AHs, markedly so in the cases at <1.5 ATA oxygen (approximately at 1/5th the AHs of the first episode).

Two comparisons were performed on the chronic CNS OT cases: (1) First episode in children (107 ± 94 AHs; case #49 omitted from calculation per above) vs. adults (117 ± 106): found to be non-significant (*p* = 0.72), and (2) Delay to treatment, based on start of continuous treatment that resulted in CNS OT. Subacute, ≤ 3 months (Cases #27, 48, 50, 57, 58: 21.1 ± 8.8 AHs), were compared to chronic cases, > 3 months [all other cases in Supplementary Tables S1-S4, except case #49 (46 cases)]: 123 ± 102 AHs, *p* = 0.035.

## 4 Discussion

Using well-identified SS of acute CNS OT, 52 cases of chronic CNS OT from outside and internal sources are presented in this study. For comparison we also gathered 7 cases of acute CNS OT from ≤5 HBOTs. These 59 cases have demonstrated that: (1) The historical rectangular hyperbola of pressure/time continuous O2 exposure for CNS OT in normal people does not apply to patients with acute or chronic neurological conditions, (2) Acute CNS OT can occur at <2.0 ATA oxygen, (3) Chronic CNS OT exists and can occur at non-traditional lower pressures (<2.0 ATA oxygen and < 100% FiO2); (4) CNS OT can occur in chronic neurological conditions at very low doses, such as compressed air at 1.3 ATA, < 100% FiO_2_ at 1.3 ATA, and even 1.15 ATA of compressed air in both portable and hardshell chambers; (5) Both acute and chronic CNS OT at <2.0 ATA occurred at 103–116 AHs, although there was wide variability consistent with the historical finding of acute CNS OT at higher pressures; (6) There was no difference in sensitivity between adults and children, but subacute intervention resulted in earlier CNS OT than delayed intervention; and (7) CNS OT was reversible if recognized early, but resulted in permanent morbidity and mortality if treatment persisted.

All cases were separated initially by pressure dose of HBOT at first occurrence of OT (cases at 1.5, > 1.5, and < 1.5 ATA oxygen and with less than 100% FiO_2_ oxygen) based on their historical presentation in our initial clinical practice and then an IRB-guided study on HBOT in chronic neurological conditions using 1.5 ATA oxygen. We found that this historical separation was arbitrary. While there was a wide range of oxygen sensitivities at a given oxygen pressure consistent with Donald’s findings (19, 20), chronic CNS OT occurred at a nearly equal average cumulative oxygen dose (116, 103, and 114 AHs), regardless of oxygen pressure. Many of these cases (#‘s: 8,9,11,12,13,14,15,17,18,20,21,22,23,25,27,34,37,39,40,41,43,44,45,46,48,50,52,53,55,59) occurred after prolonged courses of treatment and/or from intensive twice/day or 6–7d/week schedules in monoplace chambers that commonly were not equipped or did not use air breaks, a well-proven method for avoiding oxygen toxicity (48–51). Twenty-one percent occurred after an increase in oxygen or pressure dose (case #‘s: 22,27,39,40,41,42,46,51,52,53,57). This was even manifest at very low doses such as pressurized air where CNS OT was experienced after increases in chamber pressurization time to 2–3 h. Regardless of the dose at which CNS OT occurred, the CNS OT was mitigated in some cases (Case #‘s: 17,19,22,46,53,55,56) by a decrease in oxygen exposure (decrease in intensity of schedule or reduction in dose) similar, again, to the well-proven effects of air breaks (48–51).

Our study also found that repetitive episodes of CNS OT occurred at lesser doses of oxygen consistent with animal studies which have shown sensitization to CNS OT after an initial episode (52). For the cases at 1.5 and > 1.5 ATA oxygen the second episode of CNS OT occurred at similar amounts of additional AHs, 81 and 67, respectively, and for those at 1.5 ATA the third or greater episode of CNS OT after 25 AHs. At >1.5 ATA there was only a single case of 3 or more episodes of CNS OT and so did not afford a comparison to the other oxygen pressures. At less than 1.5 ATA oxygen the second episode was experienced after 22 additional AHs, and the third or greater episode after 5.4 additional AHs. These lower numbers suggested an idiosyncratic hypersensitivity, reflecting Donald’s findings of wide idiosyncratic variability in oxygen sensitivity (19, 20).

Our study identified one case (case #60) of an habituation effect to HBOT where shortly after cessation of a prolonged intensive course of HBOT there was a notable deterioration in the patient’s condition to a level worse than the pre-HBOT level. This phenomenon was characteristic of a withdrawal syndrome seen with habit-forming drugs (53). Unfortunately, there was no long-term follow-up information on this case.

If CNS OT was recognized early and accommodated no significant lasting clinical injury was apparent. This comports with the widespread clinical impression of no harm from even the most severe form of CNS OT, seizures (1, 20, 54). However, Bert (5) believed that a toxic chemical substance was responsible for the OT seizures and Bean and Siegfried (46) conceived that the toxic substance of “transient or minute quantity” might still induce tissue injury (46). This transient toxic substance of minute quantity is now known to be reactive oxygen species (2, 55). ROS/oxidative stress have been shown to occur with single HBOT exposures at both 1.5 and 2.4 ATA (56). ROS and their by-products in OT seizures in animals have demonstrated permanent effects, including lipid peroxidation (3), apoptosis (57), cognitive injury (58), motor deficits (46), and ischemic lesions (11). Similar to Bean and Siegfried’s description (46), if oxidative stress continued in the setting of SS of CNS OT lasting injury occurred. In our series this included two deaths and in a third case who required institutionalization due to severe behavioral deterioration.

Bean and Siegfried (46) methodically investigated both acute and chronic CNS OT in animals, using repetitive (3–4x/day), multi-day 5.42 ATA oxygen exposures. Their findings included: (1) OT reactions during decompression and post-decompression could be described by acute and chronic phases, (2) Acute toxicity occurred dominantly during decompression or post-decompression and was idiosyncratic with a wide variation in individual susceptibility, (3) The intensity and duration of acute reactions showed wide variation and recovery was rarely instantaneous or immediate, (4) The susceptibility to seizures was increased by successive exposures and the time to occurrence of premonitory symptoms decreased with repeat exposures, (5) Successive exposures increased the severity and prolonged the duration of the acute post-decompression reactions such that lengthening the surface interval or stopping the exposures for a day or more was necessary for recovery, (6) Seizures began most commonly during decompression, (7) Individual sensitivity to the acute and chronic manifestations was unpredictable and empiric, (8) Acute decompression effects eventually merged with chronic effects which developed gradually and upon which repeat acute reactions were layered, (9) The outstanding feature of the chronic phase was motor manifestations that were permanent, (10) Augmentation of chronic effects could be induced by increasing the duration of the oxygen exposure or decreasing the surface interval, (11) In less susceptible animals the surface interval was important: increasing the frequency from 3 to 4x/day hastened the onset and decreased the total number of exposures necessary to bring about chronic alterations, (12) A few animals that were resistant to seizures became lethargic and anorexic, (13) The reactions to O2 were generally reversed by lowering the oxygen pressure or returning to normal atmospheric pressure.

Nearly all of the above findings of acute and chronic effects of OT are exhibited by the patients described in our study’s clinical vignettes. The character and development of these patients’ adverse SS are described by finding #‘s 1, 2, 3, 4, 5, 7, 8, 10,11, 12, and 13. The primary differences between our cases and Bean and Siegfried’s (46) animal cases are: (1) the initial manifestations were often more subtle, likely due to the lower doses of HBOT used, but accumulated with repetitive exposures, similar to what has been described in large reviews of patients developing oxygen toxicity seizures during courses of HBOT for standard indications at higher doses (31–36), (2) seizures occurred both during the treatment and in-between treatments, and (3) the outstanding features of the chronic phase were not only motor, but behavioral, emotional, affective, and other. While not tested by Bean and Siegfried (46) we also found no difference in development of chronic CNS OT between adults and children.

In addition, all of our study’s cases had neurologic comorbidity, implying an increased sensitivity to CNS oxidative stress with neurologic pathology. This sensitivity was more apparent in the patients with subacute neurologic injury where OT occurred at only 20% of the first episode cumulative AHs of patients with >3 months post injury. It suggests an augmentative effect of ROS on recently injured/inflamed neural tissue. The sensitization effect of neurologic comorbidity on CNS OT is consistent with the major review by Heyboer et al. (35), however, our cases occurred at a greater average cumulative oxygen exposure, 111 AHs, than the cumulative oxygen exposure (61 AHs) for the most severe form of oxidative stress, CNS OT seizures, in six large series of patients with mostly chronic HBOT wound indications treated at the typical 2.4 ATA/90 min (31–36). This is likely due to the higher pressures used in these series. These pressures and durations of exposure are near the inflection point in the rectangular hyperbola (30) of pressure/time continuous exposures for increased manifestation of CNS OT. Oxidative stress is proportional to pressure and time of exposure with mitigation by air breaks and surface intervals. With these greater pressures there is an increased level of oxidative stress with each treatment and cumulatively greater levels of oxidative stress require greater times to decay in both CNS (46) and pulmonary OT (1, 9). Given the similar air break (surface interval) of 22–23 h in a once/day treatment in most of our and the large series’ cases it is likely that there is retained injury from oxidative stress with these higher pressure treatments that has not been mitigated by the time of the next daily.

TREATMENT. This would be consistent with the kindling effect of repetitive exposures to hyperbaric oxygen (10) and the autocatalytic nature of oxidative stress (49) where these repetitive higher pressure exposures likely reach the auto-catalytic threshold sooner than patients exposed to the lower pressures of oxygen. This, again, reinforces Bean and Siegfried’s (46) proposal of transient effects of HBOT exposures and oxidative stress as well as permanent (and cumulative) effects which have been validated by others (10, 12).

Neurologic comorbidity in our subjects may also explain the most important finding of our study, that the rectangular hyperbola pressure/time CNS OT graph with its 2.0 ATA oxygen asymptote does not apply to patients with cerebral disorders receiving repetitive exposures to HBOT. The rectangular hyperbola misled us and the medical community into the false notion that CNS OT could not occur under 2.0 ATA oxygen. The central flaw in this notion was that the curve was generated from continuous hyperbaric oxygen exposures to normal people, mostly young male military members. We speculate that the human central nervous system in normal young males has the anti-oxidant capacity to equilibrate the oxidative stress of a continuous exposure to oxygen at less than 2.0 ATA, but not above. We further speculate that had seminal researchers in CNS OT (18–20, 22) exposed neurological patients to the same continuous exposures a typical hyperbola with both y and x axis asymptotes would have likely resulted. This is what our data describes with CNS OT occurring across a range of oxygen pressures from 2.0 to 0.24 ATA oxygen which approaches an x axis pressure asymptote. The only difference is that the autocatalytic threshold appears to be in the range of 103–116 AHs of cumulative oxygen exposure in chronic neurologic patients and far less with subacute/acute neurologic disease.

In one case CNS OT was captured on functional brain imaging that tracked the clinical manifestations and recovery (Figure 1). The acute manifestations were consistent with what has been identified with CNS OT in animals, increased brain blood flow prior to seizures (59). Despite the clinical improvement in the patient, the final imaging suggested negative residual effects with areas adjacent to the toxic areas showing significant reductions in blood flow below normal. Interpretation of this finding is difficult since the area of toxicity (high activity) would be expected to be the area showing OT injury (*vide supra*), not the adjacent areas unless there is an ischemic blood flow steal phenomenon from the adjacent areas to the high flow areas during the toxicity period.

An unexpected and derivative implication of the similar SS of CNS OT regardless of pressure and FiO_2_ of oxygen is that increased pressure and hyperoxia are bioactive across the range of pressures from 0.24 to 2.0 ATA. This bioactivity, particularly for the patients who expressed SS of CNS OT at 1.3 ATA of compressed air, is additional proof that subjects receiving compressed air cannot be a control group in hyperbaric medicine clinical trials. The claim in HBOT cerebral palsy studies (60, 61) and mTBI PPCS studies (62–65) is that compressed air control groups are placebo groups. A placebo must be inert (66). If compressed air was inert it would be impossible for the subjects in our study to have experienced CNS OT.

### 4.1 Caveat

This study requires comments on the secondary issues that generated consternation in the review process and that are certain to cause consternation in some readers. Documentation of CNS OT in our cases necessitated presenting the case histories of the patients. These histories frequently reported symptomatic improvement of chronic neurological conditions before the manifestations of CNS OT and neurological deterioration. Accepting the toxicity events implies a concomitant tacit acceptance of the reports of neurological/cognitive improvement in the patient’s disorders, all of which were off-FDA label diagnoses and nearly all of which were treated with atypical pressures, durations, and numbers of treatments. This study was not intended to be an indirect endorsement of off-label use of HBOT. It intended to report a series of inexplicable clinical observations that at first were unrecognized then later appreciated as reproducible/common. These clinical phenomena contradicted decades of an unchallenged tenet on the impossibility of CNS OT at less than 2.0 ATA oxygen. They refuted not only this false tenet, but exposed the flawed confused foundation of the modern field of hyperbaric medicine (67) and the atypical, idiosyncratic, and arbitrary FDA-“approval” process applied to HBOT. The flawed foundation and FDA- “approval” of HBOT has led to the resurrection and expansion of 300 previous years of “off-label” use of HBOT seen in our cases and with the proliferation of home hyperbaric chambers.

FDA-“approval” or FDA-“label” (clearance of a drug or device for marketing in the United States) is synonymous with “scientifically proven” and “off-label” with “unproven” and “unscientific.” FDA- “approval” was historically predicated on randomized clinical trials. Not so for HBOT. In 1979 the FDA grandfathered “approval” of 13 diagnoses (68) for HBOT (the FDA-“label”) based on a Delphi Consensus of opinion (67) of a group of hyperbaric physicians that were members of the Hyperbaric Oxygen Therapy Committee of the Undersea Medical Society, now the Undersea and Hyperbaric Medical Society (UHMS). While not without clinical evidence 12 of the 13 diagnoses had no randomized trials to support their use (67), 7 of the 12 still do not, and two of the last three diagnoses added to this list by the UHMS and endorsed by the FDA without formal application similarly have no randomized trials to support their “approval” (67). One of these three, intracranial abscess, was “approved” based on 20 worldwide cases, 14 of which were reported in a case series and the additional six from a medical society call-out over 5 years for treated cases (69). The second, central retinal artery occlusion, was also approved without a single randomized trial (67, 70). In a meeting with this author in 2/2002 on behalf of the International Hyperbaric Medical Association to request evidence requirements for new FDA- “approved” HBOT indications the Devices and Radiological Health Section of the FDA admitted that the 13 indications lacked clinical evidence. To this day the main problem with new FDA “approved” indications is their dependency on the same Delphia Consensus of the UHMS HBOT Committee where the flawed definition of HBOT (67) precludes acknowledging the scientific proof for a number of the off- label indications seen in our study. The result is off-label use for some indications that have greater proof than most of the existing 15 indications (60, 71–73).

The flawed unscientific historical definition of HBOT declared that HBOT was the use of 100% oxygen at >1.4 ATA for a list of “certain recalcitrant, expensive, and otherwise hopeless medical problems” (74) determined by Delphi consensus. “Certain,” however, has led to a similar Delphi Consensus list of 23, 48, and 20 diagnoses in Russia, China, and Japan (67). Any pressurization under 1.4 ATA with 100% oxygen or < or > 1.4 ATA with less than 100% FiO2 was undefined and not hyperbaric oxygen. This begged the question, “What was it?” This entire excluded pressure and FiO2 range was the domain/basis for the 300 previous years of use of pressurized air therapy, including the introduction of hyperbaric medicine to the U.S. during the Spanish Flu Pandemic where pressurized air was used to resuscitate dying Spanish Flu patients (75). It was the bioactivity of pressure and hyperoxia along the spectrum of pressures and FiO2s > or < 1.4 ATA and < 100% O2. In particular, the bioactivity of <1.4 ATA 100% oxygen was considered by the clinical hyperbaric medicine field to be non-existent such that it could serve as a placebo control in hyperbaric studies. Supplementary Table S4 cases in this study refute this placebo control notion by demonstrating CNS OT at less than 1.4 ATA 100% oxygen, i.e., bioactivity of pressure and oxygen <1.4 ATA. If treatment in these pressure ranges were a placebo they could not generate toxicity effects. The fact that they did, consistent with the CNS OT SS at much higher doses, reaffirmed this bioactivity and refuted the non-scientific definition of HBOT.

In 2011 the FDA elucidated the flawed definition of HBOT by identifying HBOT as a dual component therapy consisting of increased pressure and hyperoxia (76). Their declaration was confirmed by 50 years of elegant physiologic studies that demonstrated the ubiquitous sensitivity of all living organisms to barometric pressure (77). Unappreciated by the clinical hyperbaric medicine field a critical review documented biosensitivity to barometric pressure in the 1.0015 to 1.3 ATA range, the identical range of the portable chambers that have proliferated in the past 20 years (77). This literature reinforced the FDA’s classification and a scientific understanding of hyperbaric oxygen therapy for the first time in 347 years.

The FDA understanding also informed the proliferation of off-label use of HBOT that began in 2001with a series of randomized “controlled” HBOT studies in acute stroke (78) and cerebral palsy (60) (CP). Rusyniak et al.’s (78) acute stroke “control” group received 1.14 ATA oxygen (a purported inert placebo control because it was outside the traditional definition of HBOT) and achieved statistically significantly better outcomes than the 2.5 ATA oxygen group, outcomes identified as “excellent” by multiple prominent stroke researchers (79). In Collett et al.’s (60) CP study 1.3 ATA air was used as a control treatment and achieved equal statistically significant improvements compared to the 1.75 ATA oxygen group in a placebo- insensitive objective measure, Gross Motor Functional Measures. Both groups also experienced cognitive improvements that have never been seen with other therapies for CP. The HBOT improvements in GMFM exceeded the improvements in GMFM for all traditional therapies for CP except dorsal rhizotomy of the spinal cord (72). HBOT was declared ineffective due to the flawed definition of HBOT identifying the lower dose HBOT groups as placebo control groups. However, the scientific basis for the positive data in these “control” groups was reinforced by a series of low pressure low FiO2 animal and human studies performed in Japan (80), while the definition-based design flaws were later reproduced in a series of U.S. DoD mTBI studies beginning in 2013 that repetitively used 1.2 and 1.3 ATA air groups as placebo controls (62–65). When the pseudo-control groups experienced equal or greater large effect size (81) symptomatic and cognitive improvements compared to the higher pressure oxygen groups the studies were again concluded to show the inefficacy of HBOT based on the flawed definition of HBOT. A scientific systematic review of these studies according to pressure and hyperoxia dosing of HBOT in mTBI has refuted these erroneous conclusions (71). The lay public, acting on these studies and responding to aggressive marketing by portable chamber manufacturers, began experiencing the results achieved by Collet in CP (60), Japanese researchers (80), the DoD TBI studies (62–65), and others (82) in pediatric and adult neurological disorders. It is this data backed by the science that has fueled off-label use and the proliferation of portable chambers for “scientifically unproven” off-label uses. Of the 60 cases in this study 30 (9 CP, 17 TBI, and 4 stroke) are for the “scientifically unproven” uses investigated in the above randomized trials. An additional 9 patients (5 with decompression illness and four with chronic carbon monoxide poisoning) were treated with HBOT based on clinical findings evident in the treatment of patients with these acute and subacute “approved”/“proven” indications. In essence the off-label use of HBOT in the U.S. is the lay public responding to the scientific evidence of bioactivity of pressure and hyperoxia in published studies and seeking treatment of their conditions based on this science. Because of the absence of an FDA label and inconsistency with the flawed definition of HBOT they remain “unproven.”

Irrespective of the science and non-science, the second major issue of this caveat is that HBOT is a prescription medical treatment that can and has been abused. Currently, anyone from a businessman to massage therapists in “spas” can purchase a hyperbaric chamber either used or on prescription from a hyperbaric chamber company physician who sight unseen will provide the prescription to any untrained person without oversight. The disturbing deaths of two children in this study and institutionalization of an adolescent from overtreatment were avoidable. They resulted from placement of hardshell chambers in homes where they were operated by untrained parents. As can be seen in Supplementary Table S4, however, complications also occurred in portable chambers at lower pressure. A hyperbaric chamber has a reasonable safety profile when used properly, however, it is not without risk, especially when high FiOj is used. While benefit seems to be accruing from expanding clinical studies and widespread public use of chambers the true incidence and prevalence of chronic CNS OT from HBOT is unknown and likely suppressed by factors peculiar to this genre of treatment. Many of the cases in this report were communicated to the authors as a sole outlet for questions regarding the poor outcome of their family member. Acting on published data, many of the families sought HBOT over objections from their primary physician or neurologist. After a poor outcome occurred there was often no physician at the hyperbaric facility to consult or physician-associated institution to whom to register a complaint. Businessmen/owners of the facilities often told the families that the outcome was inexplicable, that “everyone normally gets better.” In one case the North Carolina owner of the clinic threatened the mother with legal action and removed her from the facility’s online chat room over her complaints of new onset seizures of her child in the chamber. Family members or parents felt that they took a risk and the consequences were theirs. Case in point was a drowned child who died in a Florida hyperbaric chamber in 2020. The child’s mother lamented on social media that “I failed him (my baby), I will blame myself forever, I took the risk” (83). Safe use of hardshell chambers by the lay public has been accomplished in the UK (84) where centers were originally operated by the lay public under a non-profit trust with now de- prescriptioned 100% oxygen. It is intended that the descriptions of CNS OT in this study’s clinical vignettes will inform the medical and non-medical community of more appropriate dosing of HBOT in chronic neurological disorders and avoid the avoidable complications documented in this report. It has done so for us and allowed more nuanced and personalized treatment of patients.

In conclusion, this research study has satisfied the two purposes of research, it has answered a question and posed more questions for future research than it answered. Recommendations based on this study are for continued expanded research of HBOT indications, acceptance of the understanding of HBOT as a dual-component therapy with bioactivity across a wide range of pressures and FiO2’s, and for delivery of this therapy under physician oversight and guidance as a practice of medicine. We do not recommend the use of rote protocols for any diagnosis since medicine outside of experimental protocols is not protocol-driven, but rather individually delivered, as it has been for thousands of years. It is the use of rote protocols that has led to the widespread use/abuse of this therapy by non-medical professionals and many of the complications compiled in this report. Those complication occurred from lack of recognition/ignoring deterioration in the patient’s condition while under HBOT treatment (no medical training or expertise) and recommending extended treatment times in the chamber based on the myth that “more is better.”

## 5 Limitations

Limitations of this study include its retrospective nature and inability to verify cases from outside sources with medical records or direct observation as in the authors’ cases. However, verification was discounted due to the context of the cases: parents of special needs children are keen observers, very familiar with their childrens’ neurological deficits, and the parents alarm occurred in the setting of expected beneficial outcomes. Deviations from these expectations were easily observed. Some data is missing and details of “neurological,” “cognitive,” “symptomatic,” and “physical” “improvements” are inexact in some instances. These omissions and inaccuracies were felt to be minor because the most important part of the vignettes were the patterns and negative SS exhibited by the patients which were similar. The patterns were either a neurological reversal after neurological improvement or development of SS of CNS OT identified for acute CNS OT that worsened as HBOT continued. Both the neurological reversal and CNS OT SS could not be attributed to any other cause and they recovered after discontinuation of HBOT, similar to acute CNS OT.

Lack of objective measures of oxidative stress/toxicity is a major limitation. Documentation with biomarkers would have strengthened the study. Only one instance of biomarker documentation was serendipitiously captured with SPECT brain imaging (Figure 1). Before OT biomarkers were available CNS OT was documented by observation of signs and reporting of symptoms. The list of these identified SS are very broad and amount to oxidative stress at almost any anatomical site in the CNS. The absence of biomarkers in this study is a distinct flaw, but one that is intrinsic to the historical habit of hyperbaric CNS OT documentation. Given the non- prospective nature of this study documentation with biomarkers would have required pre/post sampling which was impossible in a retrospective study. While many biomarkers of OT are available today (85–90), none are routinely used in clinical hyperbaric medicine practice, and the results are mixed in their use in clinical hyperbaric oxygen studies (91). Future clinical studies on hyperbaric CNS OT should employ biomarkers.

Other limitations include a possible contribution of increased barometric pressure and oxygen vasoconstrictive effects. A contribution of increased barometric pressure to what has always been assumed to be the effects of oxygen pressure alone are possible. Even slight increases in pressure are bioactive for nearly all living organisms (77), but any contribution to the observed SS in our cases is impossible to parse or assess in our study. Oxygen vasoconstrictive effects might explain some or all of the CNS OT SS in acute cases 4, 6, and 7 of Supplementary Table S1, but would seem unlikely in the 52 chronic cases where ischemic deterioration would be expected in the chamber on each HBOT. This is not what we observed or families reported.

## Data availability statement

The original contributions presented in the study are included in the article/Supplementary material, further inquiries can be directed to the corresponding author.

## Ethics statement

The studies involving humans were approved by the Louisiana State University School of Medicine Institutional Review Board. The studies were conducted in accordance with the local legislation and institutional requirements. The ethics committee/institutional review board waived the requirement of written informed consent for participation from the participants or the participants’ legal guardians/next of kin because It was a retrospective chart review and due to the time over which the records were reviewed it would have been impossible to contact all of the subjects. The research would have been impossible to perform without the waiver.

## Author contributions

PH: Conceptualization, Data curation, Formal analysis, Funding acquisition, Investigation, Methodology, Project administration, Resources, Supervision, Validation, Writing – review & editing. SR: Data curation, Methodology, Project administration, Resources, Writing – original draft.

## Footnotes

^1^GraphPad.com

^2^https://www.calculator.net/standard-deviation-calculator.html
