# Supplementary material for: Acute and chronic central nervous system oxidative stress/toxicity during hyperbaric oxygen treatment of subacute and chronic neurological conditions
Source: Front Neurol. 2024 Mar 4;15:1341562. doi: 10.3389/fneur.2024.1341562 (PMC10946424; doi:10.3389/fneur.2024.1341562)
Supplement: Supplementary file 1 [file Data_Sheet_1.pdf]

Supplementary File 1: SPECT Brain Imaging in Figure 1

SPECT was performed at a university medical center on a dual-head Vertex ADAC gamma camera using 20 miCi (740 MegaBecqueres ^{99m}Tc-ECD (ethyl cysteinate dimer, Dupont Neurolite^{R}) IV in a low light quiet room in the supine position, eyes open. Image acquisition was 30 minutes after injection. Images were reconstructed using filtered backprojection with a Butterworth filter followed by attenuation correction and transferred to a SUN Sparcstation 20.^{TM} All seven patient scans were coregistered to a mean within-subject image then to a group mean image of 5 patients in a previous study. Images were spatially transformed[44] to the standardized stereotactic space of Talairach and Tournoux[45] using statistical parametric mapping (SPM) and smoothed with a 12-mm isotropic Gaussian filter. Voxel by voxel analysis was performed on individual images which were compared to a reference data bank of 55 normal subjects,mean age 34 +/- 9.5 years. Age and sex were covariates of confound. Global intensity correction was done using scaling. SPM Z maps of regionally significant differences were generated using a significance threshold for intensity of $p > 0.001$ and a Bonferroni corrected spatial extent threshold of $p > 0.05$. Thesholded maps of Z-scores are displayed with significant data as maximum intensity projections on a “glass brain” orthogonal display.
