# Supplementary material for: Acute and chronic central nervous system oxidative stress/toxicity during hyperbaric oxygen treatment of subacute and chronic neurological conditions
Source: Front Neurol. 2024 Mar 4;15:1341562. doi: 10.3389/fneur.2024.1341562 (PMC10946424; doi:10.3389/fneur.2024.1341562)
Supplement: Supplementary file 2 [file Data_Sheet_2.pdf]

SUPPLEMENTARY FILE 2: CASE HISTORIES

Table 1. Acute Oxygen Toxicity Cases:

1. The patient is a 32-year-old marine biologist with cerebral decompression illness who was treated with acute standard recompression and discharged to return to work. Due to a variety of cognitive complaints the patient was unable to return to work and 30 days post injury presented with work disability to a university hyperbaric medicine department. The author was consulted and recommended 1.5 ATA 100% FiO2/60 minute treatments, but was then reconsulted by the referring physician due to deterioration of the patient during each HBOT. Toward the end of each of the first three treatments the patient experienced pallor, weakness, nausea, vomiting, and diaphoresis in the chamber. The author discovered that the patient was being treated with the other wound patients at 2.0 ATA with 75% FiO2 to give 1.5 ATA oxygen at depth, but the treatments were two hours long. Total dose of oxygen was 3 AHs. Shorter treatments were recommended. The patient was locked out of the chamber at 60 minutes for each treatment, and she completed 24 additional treatments, cognitively improved with no adverse signs or symptoms, and returned to work.
2. The patient is a young boy with a chronic seizure disorder who underwent HBOT at a clinic with no healthcare professional staff. The patient experienced a marked increase in seizure frequency by the 5$^{th}$ HBOT at 1.75/60, bid, 5-6d/week (8.7 AHs) and had an in-chamber seizure on the 20$^{th}$ HBOT. Despite the worsening of seizures HBOT continued to approximately 40 treatments. The patient's mother continued to complain to the owner-operator who insisted that the increased seizures were unrelated to the HBOT. The patient's mother was threatened with legal action by the owner's attorney son, removed from the facility chat room, and discontinued treatment. The primary author was consulted regarding the unexpected outcome. Subsequent seizure status was unknown.
3. The patient is a 2-year-old boy, 5 months post drowning with spastic quadriplegia who underwent HBOT at 1.75/60 x 1, 2.4 ATA/60 x 1, 1.75 ATA/60 x 17, and 2.0 ATA/60 x 1. The mother reported that on the 2.4 and 2.0 ATA dives her son "cried a lot as though he was in a lot of pain and was very stressed." "While he had the hbo and particularly when they went to deep he would shake uncontrollably like he had clonis!! He never did this before starting hbo and since stopping in February he hasn't done it again." Since initiating HBOT he "developed terrible shaking in his legs and chin and arms…" Total dose of oxygen was 4.1 AHs at symptom onset. Despite the continued shaking through 20 HBOTs the child improved neurologically. The clonus abated after HBOT ended, but timing was unclear. In an email from the mother four months after HBOT there was no clonus or shaking.
4. The patient is a 44-year-old man who began HBOT bid at 1.5/45 four years after anoxic brain injury. The second treatment was at 1.75/60, immediately after which he became incoherent and couldn't eat. The third HBOT that same day was at 1.75/120 after which he was acting like "he was under the influence." The next four HBOTs were at 1.75/60 bid during which he lost his balance, gait, was falling, needed assistance of 3 people to ambulate, and lost all neurological gains he had made in the previous 2 years. Total dose of oxygen was 2.9 AHs at symptom onset. The author was consulted about this patient and a lower dose was recommended, 1.5/45 HBOTs. Two of these were performed

followed by one HBOT at 1.5/60. There was no further deterioration, but the patient's mother and referring physician stopped treatment. The patient remained in his deteriorated condition, unable to swallow, eat, or walk due to deformity and spasms of his feet. He was now feeding tube dependent. Six months later there was little improvement. Mother noted that the patient had a lifelong history of extreme sensitivity to prescription drugs.

5. The patient is a 15-year-old boy with a seizure disorder for 10 years (Rasmussen's Encephalitis) who began HBOT bid, 6d/wk, at 1.7/90 for 23 treatments. On the first HBOT, the child hyperventilated at 15 minutes, seized, vomited, was /post-ictal for 15 mins while HBOT continued. At 45 mins., hyperventilation, nausea, and vomiting recurred and the treatment was immediately terminated. In the ensuing treatments activity level and alertness increased to some degree, but seizures reproducibly occurred 20 minutes into each treatment. On the 14th treatment the facility started giving an air break 20 minutes into the HBOT. Seizures decreased over the next two treatments then increased again in the chamber. At the 19th HBOT the HBOT was decreased from bid to qd and the pressure was decreased to 1.5 ATA. Seizures became more severe and frequent in the chamber and HBOT was aborted after the 23rd HBOT. Total oxygen dose was 0.43 AHs at symptom onset during 1st HBOT. Two weeks after the last HBOT the patient went into status epilepticus, required hospital admission, and two weeks later underwent functional hemispherectomy (partial frontal and temporal lobectomies) which caused a hemiparesis and worsened expressive aphasia. He had eight subsequent seizures in the next 6 months. In the subsequent two years the patient experienced only focal facial seizures, but showed cognitive improvement with carnitine supplementation.
6. The patient is an adult male diagnosed with Amotrophic Lateral Sclerosis and TBI who began HBOT at 1.75 ATA/60 mins. qd. After 3 HBOTs the patient demonstrated symptomatic improvement. The dose was increased to 2.0 ATA/60 for one treatment, during which the patient deteriorated with drooling and decreased speech ability. Total oxygen dose was 7.25 AHs. The dose was lowered back to 1.75 ATA for two additional treatments, but there was no change in the drooling or decreased speech.
7. The patient is a 95 y.o. male who suffered a stroke at 74 years of age. A hardshell hyperbaric chamber was installed in his home two weeks later by his physicians and HBOT began for short-term memory deficits. The patient received over 3,000 HBOTs in the next 21 years at 1.35 ATA/60, qd, 6d/wk., with recovery of memory and continued academic production in a university science department. Twenty-one years after the first stroke he suffered a hemiplegic stroke. That day he received an HBOT at 1.5 for 60 mins and felt better. Approximately four hours later on the same day he received another treatment, at 1.1 ATA. Twenty minutes into the treatment he complained his head was being squeezed and had intense head pain. Total oxygen dose was 4052. He was removed from the chamber and subsequently neurologically deteriorated. Per patient request he was not taken to the hospital. Physicians were called to the house. The next day he was confused, somnolent, but still conversant. After conferring with his physicians a third treatment was attempted at 1.1 ATA and 20 minutes into the treatment he had a grand mal seizure. Total oxygen dose was 4054. Treatment was discontinued and he had 15 more grand mal seizures until his death the following afternoon.

Table 2. Chronic Oxygen Toxicity at 1.5 ATA

8. The patient is a 46-year-old commercial diver who developed worsening dizziness, restlessness, agitation during a course of HBOT after initial improvement with HBOT for cerebral decompression illness (31 treatments at 2.0, 2.4, and 1.5 ATA/90: 91 AHs). These symptoms improved over the next five months, but additional treatment was sought for residual dizziness, imbalance, and severe cognitive impairment (diagnosed with dementia after formal neuropsychological testing and neurological consultation). He was brought to the author's emergency room and hyperbaric department by his brother and attorneys after the patient was intercepted on his planned homicide of diving company management personnel.[43] The author initiated bid, 7d/week, 1.5/90,1.5/60, and qd 1.5/60 treatments without air breaks in a monoplace chamber over the course of the next two months. The patient experienced progressive improvement in his cognitive symptoms, dizziness, and imbalance. As he approached a planned 80 treatments he experienced a regression in his improved neurological condition manifest by dizziness, restlessness, and increased agitation. During the 79$^{th}$ HBOT he developed extreme anxiety and agitation and demanded chamber exit. The patient exited the chamber and sprinted out of the hyperbaric/emergency department. His total dose of oxygen was 215 atmosphere-hours (AHs). He proceeded to an attempted suicide and was hospitalized the next day in a psychiatric hospital for depression. This was initially interpreted as a psychiatric decompensation due to the extreme personal duress (bankruptcy, litigation, family dissolution, wife's emotional collapse, death of a diving partner). It was the first case of this type witnessed by the author and his colleagues and occurred with a career commercial diver who was exposed to what was previously understood to be an innocuous dose of HBOT. With the accumulation of subsequent cases this case was appreciated as an oxygen toxicity reaction due to the initial improvement of the patient then partial regression of improvement with re-emergence of dizziness (particularly after chamber exit), restlessness, agitation, and finally, extreme anxiety on the 79$^{th}$ HBOT which was felt to be partly manifestations of oxygen toxicity. Repeat neuropsychological testing during the patient's psychiatric admission demonstrated significant cognitive gains and repeat SPECT brain imaging showed a global increase in brain blood flow, both compared to pre-HBOT testing.
9. The patient is a 4 y.o. boy with cerebral palsy due to a birth injury. Mother was post-term, primigravida, and unresponsive to ptocin. Ptocin dose was doubled and patient was born 45 minutes later, tremulous, hypothermic, and with a sub-arachnoid hemorrhage. At 4 y.o. he was non-verbal, hypotonic, unable to walk without torso and leg stabilization, and had global developmental delays. Following 103 HBOTs at 1.5/90 qd and bid, 5d/wk (blocks of 52, two-week break, 22 HBOTs, one month break, 17 HBOTs, two-week break, 12 HBOTs), he was able to walk with fingertip support for balance, had stopped drooling, had improved tone, cognition, and fine motor function. Amazed at the child's progress, the mother requested prolonged additional treatment and the primary author agreed. Two months after the 103$^{rd}$ HBOT the patient received 91 HBOTs over 4 months at the same dose, qd-bid, 5d/week, for a total of 194 HBOTs. He experienced improved alertness, understanding, fine motor function, and upper extremity strength through ~185 HBOTs. Totall dose of oxygen was 416 AHs. During the last 9-10 HBOTs he experienced a regression in function with decreased tone and fine motor ability, increased

activity level, tremor, startle, and return of drooling. Untoward symptoms resolved over the ensuing month, drooling persisted, but he experienced further neurological improvement, including limited speech. Three years later the patient received 15 1.5/60 HBOTs with cessation of drooling. After a two-week break he underwent another 19 treatments with a return of drooling. This return of drooling was felt to be a manifestation of excess oxygen.

10. The patient is a 19-year-old male with developmental delays and mild mental retardation who was evaluated 3 years post eight-month hospital admission for Guillian-Barre that was complicated by multiple ischemic hypoxic and septic events. The patient was wheelchair-bound with inappropriate social behavior/touching, cortical vision impairment, worsened cognitive function, speech impairment, and depression. He began two, 40 HBOT 1.5/60 bid, 5d/wk treatment courses, separated by a 3-week break, under the author's direction in the patient's hometown. The patient experienced significant neurological and behavioral improvement. After a 3-month break the patient continued with 40,1.5 ATA qd HBOTs, but the treatment time was increased to 90 minutes by the doctor at the facility. Again, the patient improved neurocognitively and behaviorally. Three months later another round of 40 treatments at 1.5 ATA/90 caused improvement through 14 treatments then behavioral deterioration: offensive, hyperactive, rambunctious, aggressive. Total dose of oxygen was 244 AHs. This behavior partially resolved between 28 to 40 treatments. Family noticed minimal overall neuro-cognitive improvement, but a burst of improvement in the weeks after HBOT ended. Three months later the patient undertook another 15 treatments at 1.5/90 qd. He was happy and relaxed by the 3rd treatment, became hyperactive and demanding on the 4$^{th}$ (total dose of oxygen was 309 AHs), and continued to behaviorally deteriorate up to the 15th. After receiving the mother's report of the above the author recommended decreasing the treatment time to 60 minutes. Three months later the patient received 6 daily 1.5/60 HBOTs. Through five treatments the patient's behavior improved then he became fidgety, hyperactive, and short-tempered by the 6th HBOT. Total oxygen dose was 349 AHs. Over the next 12 months, the patient received 4-5,1.5/60 HBOTs every month with continued cognitive, motor, behavioral, balance improvements and became independently ambulatory. This patient's toxicity appeared to be the result of 90 minute dive times and a cumulative dose of HBOT that became manifest after a series of 6 HBOTs late in the course of extensive HBOT.
11. The patient is a 46 y.o. male who was treated 3d post carbon monoxide poisoning for neurological and cognitive symptoms with 40 HBOTs at 1.5/90 qd, 6d/wk. He experienced partial neuro-cognitive improvement and was re-treated at the same dose with three other similarly poisoned patients four months later. From the 25th-30$^{th}$ HBOT he developed dizziness, dysphoria, visual symptoms, and nausea during the last 30 minutes of treatments and/or after exit from the chamber. These symptoms progressed to lethargy, fatigue, and increasing dysphoria over the 25$^{th}$-30$^{th}$ HBOTs. Treatment was stopped. Total dose of oxygen was 146 AHs. Given that the symptoms first appeared at depth during the last 30 minutes of treatment and immediately upon exit from the chamber it was concluded that these symptoms were oxygen toxicity and the 90-minute dive time was the cause of the toxicity. The author sought consultation from other hyperbaric medicine experts since this symptomatic deterioration was thought to be impossible at the dose of HBOT employed. Neither expert had witnessed such a

phenomenon. As a result, the author sought consultation from the hyperbaric medicine community by presenting these three patients and a fourth CO patient to the April, 1995 Gulf Coast Chapter UHMS Meeting in New Orleans (abstract available from the Library of the author).

12. The patient is a 50 y.o. male who was treated 3d post carbon monoxide poisoning for neurological and cognitive symptoms with 40 HBOTs at 1.5/90 qd, 6d/wk. He experienced partial neuro-cognitive improvement and was re-treated at the same dose with three other similarly poisoned patients four months later. From the 25th-30$^{th}$ HBOT he developed dizziness, dysphoria, visual symptoms, and nausea during the last 30 minutes of treatments and/or after exit from the chamber. These symptoms progressed to lethargy, fatigue, and increasing dysphoria over the 25$^{th}$-30$^{th}$ HBOTs. Treatment was stopped. Total dose of oxygen was 146 AHs. See additional note on preceding patient.
13. The patient is a 33 y.o. male who was treated 3d post carbon monoxide poisoning for neurological and cognitive symptoms with 40 HBOTs at 1.5/90 qd, 6d/wk. He experienced partial neuro-cognitive improvement and was re-treated at the same dose with three other similarly poisoned patients four months later. From the 25th-30$^{th}$ HBOT he developed dizziness, dysphoria, visual symptoms, and nausea during the last 30 minutes of treatments and/or after exit from the chamber. These symptoms progressed to lethargy, fatigue, and increasing dysphoria over the 25$^{th}$-30$^{th}$ HBOTs. Treatment was stopped. Total dose of oxygen was 146 AHs. See additional note on two preceding patients.
14. The patient is a 52 year old male with cognitive, neurological, and constitutional symptoms from repetitive carbon monoxide poisoning in a motor vehicle over a 3.5 year period. Two and one-half years after the last exposure he received 40 HBOTs, had a two-month break, then 33 HBOTs, at 1.5/90 bid, 6d/wk. with symptomatic improvement. Symptoms partially returned in the subsequent 8.5 months and he was re-treated at 1.5/60 bid. On the second treatment, he experienced internal jitteriness/"racing," anxiety, "nervousness" in chamber. Total oxygen dose 167 AHs. HBOT decreased to once daily with tolerance of additional 18 treatments and symptomatic improvement. Patient with subsequent rounds of 8-18 treatments at same dose every 3-5 months x 3 with progressive improvement in symptoms.
15. The patient is a 60-year old physician with neuro-cognitive deficits, 2.5 years post major stroke and 3 months post-TBI with loss of consciousness (LOC)/frontal lobe contusion/bleed, who received 40 HBOTs at 1.5/90 qd-bid, 5d/wk. The patient experienced significant symptomatic improvement and commenced a second block of HBOTs at the same dose after a three-week break. At 65 HBOTs the patient was relaxed and reporting improvement in his mentation, cognition, and motor abilities. By 75 HBOTs the patient experienced significant increase in activity/energy level ("energy to burn"), voracious appetite for reading, decreased sleep, and euphoria, followed by rapid increase in irritability, short-temper, anger, arguing, aggression, agitation, and irrational thinking/behavior in the next five HBOTs. HBOT was stopped. Total dose of oxygen at this time was 169 AHs. Oxygen toxicity was felt to be the cause. Eight to ten weeks later the patient's behavior and neurological condition returned to the peak level he achieved at 65 HBOTs.
16. The patient is a 33 year old male, over three years post injury, who was part of a group of 5 chronic severe TBI patients treated in an inpatient rehabilitation hospital under an IRB-

approved protocol designed by the author. MRI at time 0 in Figure 1 demonstrated right cerebral peduncle gliosis and bitemporal and biparietal gliosis and atrophy (right greater than left). The patient had chronic motor and cognitive deficits. Due to oxygen toxicity in previous patients with 1.5 ATA/90-minute HBOTs (the carbon monoxide patients mentioned as #'s 11-14) the treatment time was shortened to 1.5/60 mins., once/d, 5d/wk treatments in 40 treatment blocks. The patient received two blocks of 40 treatments, separated by one month and experienced neurocognitive, symptomatic, and SPECT brain imaging improvement. However, the SPECT also demonstrated an area of intensely increased flow adjacent to a frontal lobe contusion (Figure 1). Due to the clinical improvement the IRB approved a third block of 40 treatments at the same dose, despite the primary author's objections and warnings regarding oxygen toxicity in repetitive 40 treatment blocks at this number of treatments and with the present dose. The patient tolerated the treatment at first then showed a progressive deterioration with increasing agitation, aggression, bizarre, and uncontrollable behavior by 110 total treatments. Total oxygen dose was 165 AHs. By the mother's demand the patient was removed from the study. The behavioral deterioration and agitation resolved over 8-10 weeks. Repeat SPECT imaging after 110 HBOTs showed a more exaggerated focal increase in blood flow at the previously identified peri-contusional site. Follow-up 1 year later on SPECT demonstrated a reduction in the focally increased area and simultaneous reduction in regional blood flow to below baseline in areas adjacent to the toxic increases in flow (Figure 1).

17. The patient was a 4-year-old boy, 16 months post drowning, who commenced 80, 1.5/90 bid, 5d/week HBOTs in 5 months in two blocks separated by a one-month break. The patient was treated at an outside facility after evaluation with SPECT brain imaging under an IRB-approved protocol at the primary author's facility. At 20-25 HBOTs the patient developed frequent myoclonus and was "pooping out," but showed increased cognitive, motor, and tone improvements (total oxygen dose was 45 AHs). Myoclonus continued and EEG after 80 treatments was negative. Three months later while on break with decreasing myoclonus EEG showed seizure activity. The child was placed on Depakote with improvement of myoclonus. The mother returned to New Orleans five months after last HBOT and the HBOT was decreased to 1.5/60 minutes bid, 5d/week, to minimize oxidative stress. Myoclonus decreased as HBOT treatments increased so patient's mother independently discontinued Depakote at the 17$^{th}$ HBOT. The child experienced neurological improvement, but by the 32$^{nd}$ HBOT was fatigued and had 4 myoclonic jerks in the chamber (228 AHs). Depakote was resumed at this time. On the 34$^{th}$ HBOT he had two more myoclonic jerks in the chamber and HBOT was discontinued. A 1-month break ensued with resolution of fatigue and myoclonus. Discussions ensued with the patient's mother and pediatrician about myoclonus as a manifestation of oxidative stress. Knowing this, due to the child's neurological improvement with each round of HBOT, HBOT was resumed in his home state by his physician team, using myoclonus as an endpoint of treatment. The child received 31 HBOTs at the same dose and toward the last few treatments patient developed fatigue and myoclonus again (276 AHs). HBOT was discontinued. It was apparent that continued blocks of treatment bid despite the decreased dose was too much. During a four month break the myoclonus and fatigue abated and the Depakote was discontinued again. HBOT was resumed at the same dose, but qd, and myoclonus returned by the 32$^{nd}$ HBOT and was severe by the 35$^{th}$ HBOT

with a seizure later that night. HBOT was discontinued. Total oxygen dose was 326 AHs. Patient proceeded to nightly seizures and Depakote was restarted without effect. After a 2-4 month break HBOT resumed at the same dose for 10 treatments. While the patient improved and seizure frequency decreased, he became extremely irritable with frequent verbal outbursts and aggression directed to his mother. Total oxygen dose was 349 AHs. Patient was lost to follow-up.

18. The patient is a 19-year-old male who received 30 2.0/90 min. bid HBOTs in a nearby state 7 weeks after a drowning episode. He experienced improved level of consciousness and awareness, but developed severe spasticity. Nineteen months later he received 190 1.5-2.0/30-60 bid HBOTs over the next 10 months with neurological improvement then plateau. The author was consulted for further dosing, imaged the patient with SPECT before and after a 1.5 ATA/90 HBOT. Mother returned to home state and unbeknownst to the author installed a hardshell chamber in her home that was donated by a movie star, and continued treatment at home at 1.5/90 qd. Within 30 treatments the patient was independently ambulatory and had clear speech. By the 55$^{th}$ HBOT (276 total) he had "boundless energy" (465 AHs). Follow-up conversation with the author after 130 HBOTs at 1.5/90 qd (351 HBOTs total) revealed sustained neurological improvement. The primary author recommended medical oversight and careful observation based on prior experience with oxygen toxicity. This was ignored. Patient continued to receive HBOT at home. Follow-up phone conversation 1-2 year later at over 501 HBOTs total (971 AHs) total revealed progressive behavioral deterioration with aggression, anger, and irrational and uncontrollable behavior which necessitated institutionalization. Once institutionalized mother would bring patient from the facility to home for continued HBOT. Treatment was terminated after the conversation with the author.
19. The patient is an 18-month-old girl with quadriplegic CP and seizure disorder who received 40, 1.5/60 bid HBOTs, took a one-month break, then finished 37 more treatments at the same dose qd-bid. Over the next eight months, she received 13,11,12,13, and 6 treatments (total 132 HBOTs) every 6-8 weeks at the same dose, qd, with neurological and symptomatic improvement,. During the last 6 treatments she deteriorated, became "wild," according to her mother, with exacerbation of tone, and decreased appetite. Total dose of oxygen was 197 AHs. HBOT was stopped. Negative symptoms resolved over the next few weeks. Due to neurological improvements with each short course of HBOT she received 7 additional HBOTs 6 weeks after the132nd HBOT. By this time seizure frequency had increased slightly to once/month. During the subsequent 5 months seizure character and quality changed from one severe seizure/month with status epilepticus requiring ER treatment to mild, short seizures, 1x/day. In the middle of this five-month period one more HBOT was delivered and immediately afterward her mother reported that she was "wired," with hyperactivity, increased tone, and 4h sleep that night. HBOT was discontinued. Total oxygen dose was 210 AHs These symptoms lasted one day. Four months after this 140$^{th}$ HBOT and seven months after the 132$^{nd}$ HBOT seizure frequency had decreased slightly to 4-6/week. Due to the milder nature of the seizures, continued neurological improvement, yet obvious sensitivity to the previous dose of HBOT, the HBOT dose was decreased to 1.25/60 qd and the patient given 6 treatments. Some twitching in chamber occurred during the 6$^{th}$ HBOT and HBOT was stopped. Total oxygen dose was 217 AHs. Seizures had decreased during these 6 treatments and ceased over the following 6 weeks with residual

clonus on exam. Variable myoclonus and seizures (1 every two weeks) recurred over the subsequent 2 months. She then received 4 more HBOTs at the same lowered dose without incident.

20. The patient was a 3-year-old girl with athetoid CP who demonstrated neurological improvement with 80 HBOTs at 1.5/60 bid, 5-6d/wk, in two blocks of 42 and 38 treatments over 5 months. The first week home after the 80th treatment the patient was jittery/tremulous and then over the next 2 months the patient showed global clinical improvement. HBOT resumed with shorter courses of 12, 10, 10, 10, and 10 HBOTs at the same dose bid over the next 8 months with continued improvement. After a one month break she resumed bid HBOTs at the same dose and during the 3rd HBOT became extremely jittery/tremulous. Total oxygen dose was 203 AHs. HBOT was discontinued and the tremors resolved in one day. She subsequently resumed HBOT at a lower dose (1.25 ATA) for a short period without incident.
21. The patient is a 10 y.o. girl with quad CP who demonstrated neurological improvement with 40 HBOTs at 1.5/60 bid. After a month break HBOT resumed at the same dose. Further improvement was noted, but as she approached 65 HBOTs she developed lethargy, startling to loud noises, and began biting individuals. HBOT was stopped. Examination revealed diffuse hyperreflexia and clonus on exam. Total oxygen dose was 97 AHs.
22. The patient is a 3 y.o. boy diagnosed with spastic diplegic CP and suspected subcortical seizure disorder (10-20 myoclonic "seizures"/day, negative EEG) who received 37 bid HBOTs at 1.5 ATA/60 mins. Patient showed some improvement in awareness, spasticity, and arching. Coincident with the last 2 HBOTs, however, myoclonus frequency increased to 50/day and HBOT was discontinued before a planned 40 treatments (54 AHs). Increased myoclonus frequency resolved in the next week. HBOT resumed 4 weeks after the 37th HBOT at the same dose with 42 treatments qd-bid from over 6 weeks with increased alertness, head and neck control, and decreased spasticity with less opisthotonos. No change myoclonus. Myoclonus frequency decreased over the ensuing 3 years and patient sought hyperbaric treatment in their home state. He underwent a single HBOT at a higher dose, 1.75 ATA. Part-way into the treatment patient had his first grand mal seizure, followed by another seizure at home within days. Total oxygen dose was 120 AHs. EEG was negative, but the patient was started on Keppra, weaned from the Keppra months later, experienced another grand mal seizure, was restarted on Keppra, weaned again, and again experienced a grand mal seizure. This cycle continued for 5 years until the patient was without seizures for one year. Mother then reattempted HBOT at a lower dose, 1.3/55 mins. in a multi-place chamber. On the 8th treatment patient had a complex partial seizure 40 mins. into the treatment. Total oxygen dose was 129 AHs. Patient was decompressed while seizing and continued to seize for greater than 6 minutes until his hood was removed upon exit from the chamber. No Follow-up.
23. The patient is an 8 y.o. girl with a VP shunt and baclofen pump in a persistent vegetative state 3y 2mos. after a severe TBI incurred in an MVA. She was experiencing 5-10 seizures/day, some grand mal, was on three anti-convulsants, a cooling blanket for thermal dysregulation (frequent temperatures to 104/105 degrees), and was deteriorating with increasing frequency of drug resistant pneumonias, copious pulmonary secretions, and respiratory failure. The patient experienced progressive neurological improvement through 75 HBOTs bid at (unknown dose, but historically given at <1.75 ATA/60 minutes

for neurological patients at this facility. Estimated at 1.5 ATA based on father's use of this dose once he had his own chamber in home), but with increasing irritation from 40-75 HBOTs (oxygen toxicity considered at 40 HBOTs, 60 AHs). Seizures, myoclonus, and thermal dysregulation ceased, suctioning was no longer required, tracheostomy was decannulated, she started eating by mouth, was more alert and aware, responded to simple commands, and SPECT brain imaging was improved. After a two month break the patient received 30 more HBOTs bid at the same facility, unknown dose, but was stopped prematurely due to increasing agitation/irritation again. (Estimated 158 AHs, assuming 1.5/60 HBOTs). By this time all 13 medications were discontinued. Patient returned to school, the baclofen pump was removed, and spasticity was absent. Fourteen months later the patient's father installed a hardshell hyperbaric chamber in his home, delivered 15 HBOTS at 1.5 ATA/for unknown period of time, once/day, with further neurological gains then started cycles of 2-3 month breaks, 5-15 HBOTs at 1.5 then 1.3 ATA/unknown time. Eventually, the patient's seizures returned. The father read literature and was aware that the hyperbaric physician that first treated his daughter had successfully treated seizures. Since he had seen his daughter's trauma-induced seizures eliminated with HBOT he continued to treat with HBOT and the seizures progressively worsened in frequency and intensity until the patient died in status epilepticus with cardiac arrest nine months after installing the chamber at home. Estimated at least 40 HBOTs since chamber installation. Father did not keep treatment records. Total oxygen dose was estimated at >180 AHs.

24. The patient is a 7 y.o. boy. At birth he was one-week post-dates, underwent induction, had shoulder dystocia, failed suction extraction, and was delivered by forceps at 10lbs. 14oz. He required oxygen immediately. Normal development until 12 mos. when stopped following commands. At 18 mos. not "tuned in", distracted, then speech delay by 2 years old. Diagnosed autism, extensive treatments, some improvement. Begins HBOT, 1.5/60 qd x 40. Social, behavioral, speech improvement, obsession with balloons ceased. One month post treatment, behavioral deterioration, self-injurious. Dietary change two months later helpful. Ten months post HBOT-"best year of his life"-started to read, accomplished all IEP goals and had to redo IEP 5 months into school year; goals changed from behavioral/self-help to academic. Started doing math. Resumes HBOT 10 months after last HBOT, same dose, qd. By 21$^{st}$ -25$^{th}$ HBOT (61-65 total) (91 ATA-hrs.) developed mood swings, slight decrease in word-finding, increased agitation and hyperactivity. Thought to be transitional phase of treatment. Improved symptoms on weekend break, further increased agitation on next two HBOTs. Night of 67th HBOT became obsessed with balloons again and smearing feces, laughing, sliding on floor in feces (not seen in the previous four years). The following day on the way to the clinic for 68$^{th}$ treatment screamed and kicked in the car the entire drive to the clinic. Refused to go in the chamber room. Normally enjoyed treatment and excited/anxious to come to the clinic. Over next 4d agitation and hyperactivity improved. Complete regression of negative behavior by two weeks post HBOT. Two months post HBOT mother reports increased receptive language, understanding of time, abstract thinking, following complex commands. Given one additional treatment at lower dose. No followup.
25. The patient is a elderly man with Creutzfeld-Jakob Disease who was treated with HBOT at 1.5 ATA/unknown time, qd-bid. Within the first few weeks he demonstrated some improvement in his mental status, but became increasingly restless. He was administered

Celebrex daily and glutathione IV 2gms for the restlessness and experienced a 20-minute period of lucidity the first night, followed by 45 mins the following day, and then 90 minutes on the 3rd day. Total oxygen dose was estimated at ≥ 15 AHs. HBOT continued and the restlessness increased. The family decreased his Dilantin dosage substantially, suspecting this was a Dilantin side-effect. As the HBOT continued the patient became increasingly spastic over a few days and Ativan was initiated at night for the restlessness and spasticity. HBOT was decreased to once/day due to morning sedation from Ativan, but his behavior continued to degrade with periods of aggressiveness as HBOT continued. He became incontinent, asked his family to let him die with dignity (due to diaper dependence) and was transferred by air ambulance 200 miles back to home city and acute care unit to stabilize "psychiatrically."

26. The patient was a 2 yr 11-month old female suffering from a birth injury. She had one seizure at birth. At approximately 2.5 y.o. patient received 17 HBOTs at 1.5ATA/60. Treatment was terminated by an upper respiratory infection. Improvements included a reduction in spasticity, able to feed herself, get up on all four extremities, assisted gait. Approximately four months later she received HBOT at another facility at 1.5 ATA/60 mins at depth in a multiplace chamber. She was developing jerkiness in the chamber on oxygen and eventually between treatments. Twenty minutes into the 15th HBOT the child had a complex partial seizure. Total dose of oxygen was 48 AHs. A neurologist evaluated her, sleep deprived EEG revealed "spikes," and she was started on Lamictal. At 1.5 months post 32nd HBOT she had a second seizure described as a complex partial. Lamictal dose was increased, however, the jerkiness persisted then started to decrease after two weeks.
27. The patient was a 7 month old girl, one month post herpes simplex meningitis and encephalitis characterized by seizures during early part of infection, who was functionally blind, had continued tongue extrusion, social withdrawal, and passivity/lethargy/sleepiness. Patient was treated at primary author's clinic and received 7 daily 1.15/45 min. HBOTs with improvement in vision, reach/grasp, transferring objects between hands, increased activity, decreased amount of sleep. Continues to 11 HBOTs total and mother had to return to home state for personal emergency. Two days post flight home the patient has a single seizure. Restarts HBOT in home state 3d post seizure at increased dose of HBOT, 1.5/90 bid. During second, fourth, and fifth HBOT has a seizure in chamber 30 minutes into each treatment. Total oxygen dose was 12.5, 15.5, and 16.3 AHs, respectively. During final seizure in chamber mother calls primary author on her cell phone while she is in the chamber with her seizing daughter to ask him to notify personnel at HBOT facility that child is actively seizing in chamber (no attendant in room with chamber during treatment). HBOT stopped. Despite seizures mother noted clinical neurological improvement in her child, but in the days to weeks after the last seizure child develops seizures that increase to a peak of 40-50/day. Child was medicated with anti-convulsants and seizures waned over the ensuing months.
28. The patient is a 50 y.o. female with TBI and persistent post-concussion syndrome stemming from a motor vehicle accident (MVA) with loss of consciousness (LOC) 16 months pre-HBOT. Patient was evaluated in the ER and had persistent symptoms from the time of the accident. Vestibular therapy worsened her symptoms. A neurologist diagnosed her with post-concussion syndrome and her symptoms slowly improved but persisted after multiple therapies over a twelve-month period. She was entered in a

clinical trial and underwent 39 HBOTs at 1.5 ATA for 60 mins qd and experienced postconcussion symptom improvement with a 24% reduction in her Neurobehavioral Symptom Inventory. In the last week of treatment the patient felt some fatigue that increased by the 37th and 38th HBOTs and waned with a 3d break. HBOT resumed and fatigue returned to its greatest level and was associated with confusion after the 39th HBOT (59 AHs). HBOT was discontinued. The following day at the airport patient experienced an emotional crisis becoming overwhelmed by the noise and commotion at the airport. Once home she experienced increased irritability, anger, headache, cognitive deterioration. These negative symptoms resolved over the next 3-4 weeks.

29. The patient is a 49 y.o. female who sustained an mTBI without LOC from a fall. She developed a persistent postconcussion syndrome (PPCS) with fatigue, short-term memory loss, headache, sleep disruption, balance problems, and diplopia, especially going downstairs. Eight years after the accident she still complained of fatigue, mental confusion, short-term memory loss, sharp headaches every 2 wks. with nausea, light and sound sensitivity, and dizziness, had a right foot drag, and disrupted sleep. She was enrolled in a clinical trial and HBOT was initiated at 1.5 ATA/60 mins., qd, 5d/week. By the 12th HBOT patient had a 36% decrease in her postconcussion symptoms, was feeling "great, euphoric" after exit from the chamber. She then began a reversal of this improvement by the 17th HBOT with absence of positive sensations after chamber exit and transient dizziness after the 22nd and 23rd treatment. Following the 26th and 27th HBOTs the patient complained of shortness of breath and dypsnea on exertion. Peak flow pre/post the 28th HBOT showed a 9% decrease. Patient was prescribed a bronchodilator to use pre-treatment. After two additional treatments patient developed extreme fatigue, cognitive reversal, and paresthesias. HBOT was discontinued at 30 HBOTs. Onset of symptoms at 22 HBOTs was a cumulative 33 As. Over the next 19d adverse symptoms resolved.
30. The patient is a 45 y.o. female with PPCS following an MVA with LOC who was entered in an HBOT clinical trial and began HBOT 3.5y after the injury. At that time she reported constant head pressure and localized headaches, problems with vertical gaze, nausea, motion sickness, memory issues, and cognitive problems. HBOT commenced at 1.5 ATA/60 mins., qd, 5 d/wk. After the 3rd HBOT she reported mild SOB in chamber. This was repeated during the next two HBOTs, along with a feeling of inability to take a deep breath. Peak flow pre/post these treatments showed a 6-7% reduction. By the 7th HBOT no change in peak flow occurred pre/post HBOT and she was allowed to continue treatment due to the mild nature of the symptoms. At the end of the treatment course she reported that she had concealed mild SOB after each treatment that waned over hours post-treatment. This was interpreted as pulmonary oxygen toxicity. By the 32nd HBOT she started experiencing increasing fatigue and after the 36th HBOT was confused and couldn't converse. Total oxygen dose was 54 AHs. This was reported after the 37th HBOT when the same occurred again and she slept in the chamber for the first time. She also had a concomitant mild URI. HBOT was discontinued. Her fatigue and negative symptoms receded over the next few weeks with retention of symptomatic and cognitive improvements.
31. The patient is a 48 y.o. female with PCCS from an MVA with LOC who presented to the author 22 months after injury with frequent complex migraine headaches, fatigue, and cognitive symptoms that were exacerbated by altitude exposure. She underwent

extensive therapies with minimal success, finally entering an HBOT clinical trial. She underwent 39 treatments at 1.5 ATA/60 mins, qd, 5d/week. She experienced progressive symptomatic and physical improvement with Neurobehavioral Symptom Inventory scores declining by 81%. Over the last 3 HBOTs she experienced increasing fatigue with treatment which had been identified as an early sign of oxidative stress/OT in other study patients. Total oxygen dose was 55 AHs. Her treatment was discontinued. Fatigue resolved over the next 3 weeks.

32. The patient is a 38 y.o. male with PPCS after two mTBIs who entered an HBOT clinical trial 6 months after the second TBI (one year after the first TBI). He underwent 34 treatments at 1.5 ATA/60 mins., qd, 5 d/wk, experiencing progressive symptom improvement and declining scores on the Neurobehavioral Symptom Inventory (NSI) until the 29th HBOT when he noticed a return of pre-HBOT twitching. Over the next 5 HBOTs patient experienced increased sleep disruption, dizziness, fatigue, weakness, shakiness, blurry vision in his right eye, nausea, and flu-like symptoms immediately after treatments that persisted between treatments. Total oxygen dose was 43 AHs. HBOT was discontinued after the 34th HBOT. Negative symptoms progressively waned and were gone 10d after the 34th HBOT.
33. The patient was a 45 y.o. male with PPCS for 12 years after a motorcycle accident that was exacerbated by a second mTBI four years later and a third mTBI ten months before entry into an HBOT clinical trial. He received 40 treatments at 1.5 ATA/60 mins., qd, 5d/wk, experiencing symptom improvement and declining NSI scores. In the last few treatments he noticed increasing fatigue and foggy-headedness, but did not report this. These symptoms waned over a two-week period after the 40th HBOT. Total oxygen dose was 57 AHs. Final post-concussion symptom score (NSI) had decreased by 74% two months after the 40th HBOT.
34. The patient is a 3 y.o. girl with CP who underwent 40 HBOTs at 1.5 ATA/60 mins, bid, and experienced a decrease in spasticity during the first 20 HBOTs then progressively lost this improvement to nearly her baseline level of tone as she received HBOTs 20-40. Total oxygen dose at 21 HBOTs was 31 AHs. Four months later she received another 12 HBOTs at 1.5 ATA/unknown duration, bid, with minimal change and 2 months later 23 HBOTs, qd at 1.75 ATA/unknown duration with no effect. Two months later the parents consulted the primary author who recommended a reduction in pressure to 1.3 ATA. One month later the child received 18 HBOTs at 1.3 ATA oxygen/unknown duration, improved neurologically and 10d later took 10 independent steps. 2 months later 8 HBOTs at 1.3 ATA/unknown duration with slight reduction in tone.
35. The patient is a 15 year-old boy with birth injury, ADHD, autism, and PPCS from multiple mild TBIs who underwent daily HBOT 8 months post last concussion at 1.3 ATA/90 mins. at depth x2, then 1.5 ATA/ 90 mins. at depth x 25. Experienced cognitive and affective improvements, decreased agitation, increased motivation, decreased joint pains. Five months later he underwent an additional 29 HBOTs for 90 minutes each at depth beginning at 1.3, then 1.5, 1.75, one at 1.3, and last half of the 29 at 1.5 ATA. By 20 HBOTs patient experienced increasing fatigue, agitation, frustration, headache in the chamber, dizziness on chamber exit, difficult processing information, absent-mindedness, “I don’t feel like myself.” Total oxygen dose was estimated at 105 AHs. He had to take three weeks off of work. Author was called and recommended reducing the chamber time by 50% to see if symptoms would resolve. The facility treated another 9 HBOTs at

1.5/45 mins. at depth. Total oxygen dose was estimated at 135 AHs. The patient experienced a mild decrease in light sensitivity and hypersensitivity to touch, but the fatigue increased, headaches and irritability persisted. Patient finally refused to go in the chamber. After a one-month break and no change in symptoms patient was evaluated by the primary author with SPECT, single hyperbaric air (HBA) at 1.3 ATA/45 mins., demonstrating a global increase in blood flow, improvement in affect and energy level, and decrease in irritability. Two months later the patient began normobaric oxygen with 10L/minute/50 minutes, 3-4x/week then added HBA at 1.3/30-45 mins. 3-5x/week with 198 treatments in the next two years. In the second year of treatment the primary author recommended changing the dose to HBOT at 1.3/30-45 mins. with oxygen 3-5L/min by venti-mask, 3-5x/week. During these two years he was able to improve his academic skills, graduated high school, completed a trade school welding course with a certification, passed the military entrance exam he had failed multiple times, and began the military enlistment process. His PPCS symptoms of light sensitivity, headaches, anger, brain fog, and irritability resolved.

36. 51 y.o. woman with persistent short-term memory loss, sleep disruption, myalgias, body pains, and intermittent chest tightness two years post COVID infection. Six months post infection she tried HBOT with 3 treatments at unknown dose, but discontinued due to middle ear barotrauma and Eustachian Tube Dysfunction. One and one-half years later she re-attempted HBOT for persistent symptoms at 1.3 ATA/60 TDT x 1, then 1.5 ATA/60 TDT x8, qd, in 13d. On the 9th total HBOT (6th in this series of 9) she experienced slight lightheadedness. Patient was told to eat before HBOT. On the next two HBOTs experienced the same symptom despite eating before HBOT, yet also experienced improvement in her post-COVID symptoms.. On the 12th total HBOT (9th and last of this series) the patient experienced extreme light-headedness, tachycardia, diaphoresis, near LOC, feeling of doom, “felt like going to die.” She had no tinnitus, hearing loss, vertigo, or middle-ear barotrauma. HBOT terminated. Total oxygen dose was estimated at ≥ 13 AHs. Symptoms resolved in 11d except palpitations/tachycardia. Patient requested lower dose, facility refused and she was told “not our protocol.”

37. 52 year-old male, Type II decompression sickness (cord and brain), 1 U.S. Navy Table 6A Modified, U.S. Navy Table 6 x 14, 1 U.S. Navy Table 5. Residual symptoms: fatigue, cognitive complaints (memory, reasoning, intellectual function), painful spasticity and paresthesias in legs and perineum, lower abdominal discomfort, severe constipation (bowel movements q 5d), depression. Sees primary author 3y 7mos. after initial treatment and enters IRB-approved study with HBOT at 1.5 ATA/90 minutes (no air breaks) twice/day, 5d/week. By 50 HBOTs significant improvement in all symptoms, “90% back to normal,” but patient nearing plateau by symptom change and physical exam. Primary author recommended stopping HBOT, patient pleaded for chance at additional 10% improvement. Author agreed with caution. HBOT continued with some minor further improvement in temperature and touch sensation in buttocks and legs, balance, and strength in lower extremities until last week (10 HBOTs) of treatment when lower extremity spasticity and dysesthesias and constipation progressively increased. Total oxygen dose was 139 AHs. Primary author stopped treatment over patient’s objections at 72 HBOTs due to worsened symptoms. Patient went on Tegretol with significant, but incomplete relief of negative symptoms. Over the next six months negative symptoms waned and Tegretol was weaned. Patient maintains frequent

followup with primary author and at this time is 28 years post last HBOT with minimal symptoms and good quality of life.

38. Elderly male, 2 years post stroke, commenced HBOT for stroke deficits. Patient received eighteen HBOTs at 1.6 ATA, 94% oxygen from oxygen concentrator/67.5 mins. oxygen treatment time (from reaching depth to surfacing), once daily, 5d/week. Improved cognition (car-driving improving) and much more energy by 11 HBOTs, but aggressive/hyperactive on drive home from treatments. After the 18th HBOT so hyperactive could not sleep the entire night and was very agitated. HBOT stopped. Total oxygen dose was 19 AHs. Hyperactivity and agitation regressed to normal. Repeat qEEG after the 18 HBOTs showed a global deterioration with shift in absolute power to the lower frequencies, a decrease in balance (relative power), and a worsening of the connectivity metrics.

Table 3: Chronic Oxygen Toxicity at > 1.5 ATA oxygen.

39. The patient is a 58-year old man, three years post cerebral aneurysm rupture, who underwent HBOT at 1.75/60 x 3, 2.0 ATA/60 x 5, 2.5 ATA/60 x 11, 2.8 ATA/60 for 1, 2.5 ATA/60 x 5, bid, 7d/wk. Neurological improvement occurred through 19 HBOTs then he became wild, combative, confused on 20th treatment within minutes at depth. Air-break resolved symptoms, oxygen breathing resumed, patient became incoherent. Total oxygen dose was 46 AHs. No further improvement of symptoms on decreased dose of last five treatments. In the next 2-3 weeks he lost all neurological gains and experienced progressive behavioral deterioration over 2-3 months. HBOT resumed 7-8 months post last HBOT at 1.75/60, bid, 7d/wk x 40. Neurologically improved by 10-15 HBOTS, but increasing agitation at depth. 35th HBOT: aggressive, angry, fatigued, lethargic, HBOT stopped, total dose 119 AHs. The next day worsened aggression, confusion, anger, "went berserk," complete regression of neurological gains. Extensive neurological workup was negative. Three months later wife consults author, 10 HBOTs at 1.5/60 qd, 5d/week, improved behavior. Ten to fifteen days post HBOT continuous neurological improvement over 3 months to less than peak during the first course of HBOT. Three and one-half months after last HBOT the patient became incoherent and incontinent, hospital admission, extensive work-up negative, unchanged brain CT. In hospital wife administers oxygen, nasal cannula, 1L/min (no effect), 4-5L/min, patient becomes coherent, improved symptomatically, discharged home.
40. The patient is a 21-year-old male who had experienced a severe traumatic brain injury with diffuse axonal injury, subarachnoid hemorrhage, epidural hematoma, and Le Fort III fracture. Massive cerebral edema and elevation of intracranial pressures resulted in prolonged hospital stay. A year after injury the patient developed petit mal seizures which progressed to grand mal seizures one year later. Shortly thereafter he was discovered to have a frontal lobe abscess and underwent three cranial surgeries including a drainage procedure, repair of ethmoid sinus fracture, and then repeat drainage procedure for recurrence of the abscess. Seven and ¾ years later the patient proceeded to Canada for hyperbaric oxygen therapy. He underwent eight 1.5/60 bid, 6 day/wk HBOTs without improvement. The pressure was increased to 1.75 ATA/60 bid, 6 days/wk and by ten additional treatments the patient had lost his balance and ability to walk. He broke furniture and a toilet in the hotel room and became irritable and more impulsive. Total

dose of oxygen at onset of toxicity was 29 AHs. Irritability, anger, aggression, and belligerence increased by thirty treatments. He was most agitated in the chamber at depth. At 39 treatments the mother brought the patient home unable to walk, with the above behavioral/emotional problems. Total dose of oxygen was 67 AHs. In the ensuing weeks his agitation increased over a two-month period, his coordination deteriorated globally, especially fine motor coordination, as evidenced by inability to feed himself. In the ensuing nine months the patient had slight improvement in balance and agitation but was still unable to walk, was impulsive, inappropriate, and unable to feed himself properly. The patient presented to New Orleans and underwent a sequence of SPECT brain imaging, single hyperbaric treatment at a lower dose, 1.25 ATA/60, and repeat SPECT brain imaging. The second SPECT scan showed a greater than 250% increase in brain blood flow and a noticeable smoothing of the abnormal pattern. The patient underwent a course of 1.25/60 bid, 5-day per week HBOTs with progressive improvement in behavior and cognition and a decrease in impulsivity and inappropriateness. Coordination and balance were slightly improved.

41. The patient is a 33 y.o. male who undertook a 60 ft. SCUBA dive in freshwater at altitude and sustained cerebral DCI. On month later he was treated unsuccessfully with three monoplace U.S. Navy Table 6 HBOTs for dizziness, imbalance, dysarthria, ataxia, fine motor incoordination, and cognitive deficits. Four months later he underwent 40 consecutive bid 1.5/90, 7d/wk HBOTs and 40 bid 1.75/90, 7d/wk HBOTs, experiencing significant improvement in his condition, but was mildly irrational, hyperactive, and euphoric. He was diagnosed hypomanic by the participating psychiatrist. His total dose of oxygen was 211 AHs. These symptoms resolved in 2-3 months and he was treated with 15 HBOTs in 6d at 2.0/90 tid-bid for mild residual cognitive complaints. The patient reported improvement of symptoms initially, but HBOT was discontinued after the 15$^{th}$ HBOT due to dysphoria, tremor, dizziness in the chamber. (256 AHs). Symptoms resolved over the next week. Patient continued his employment, obtained two master's degrees and remains employed doing neurocognitive testing in a hospital-based TBI program at the time of writing.
42. The patient was a 9 y.o. girl, five years post-drowning, who underwent 51 1.5 ATA/60 bid, 5d/wk HBOTs with motor and cognitive improvements. Three months later, she resumed treatment in a second state at 1.5/60 qd, 6d/wk for 29 HBOTs. Seven months afterwards she received treatment in a third state at 2.0/120 qd. On the 19th treatment she seized at depth. Total oxygen dose was 196 AHs. Treatment resumed in New Orleans with the author 9 months later at the initial dose, 1.5/60 qd for 25 HBOTs. The patient improved symptomatically. She received additional treatment in a fifth state 4 months later at an increased dose (longer treatments, 90 minutes) at 1.5/90 bid, 6d/wk. On the first HBOT she improved. With each successive HBOT she became increasingly fatigued and the mother reported extreme anxiety, hyperactive movement, and an appearance of discomfort in the chamber. She completed 24 HBOTs. By the 2nd HBOT, total oxygen dose was 238 AHs. Mom tried 14 more HBOTs at the same facility on the same schedule six months later with no improvement.
43. The patient was a 4-year-old diplegic CP boy who received 22 (11 Rx's/wk) 1.75 ATA (95% oxygen)/60 HBOTs with symptomatic improvement. In the next 3 months there was mild regression of the improvements and HBOT was reinstituted at 1.75 ATA (100%

oxygen)/60 (11 Rx/wk). On the 56th HBOT (78th total) he seized at depth; he had no prior seizure history. His total oxygen dose was 135 AHs.

44. The patient is a 12-year-old quad CP boy who underwent 40 1.75/60 bid 5d/wk HBOTs, then 50 treatments in doses of 10, 20, and 20 treatments at 1, 2, and 1 month intervals. Four weeks later HBOT was reinstituted and by the 15th HBOT he experienced lost speech, decreased attention, cognition, balance, coordination, pallor, increasing lethargy, was "rubbery legged, droopy and drunk," started falling, and became wheelchair dependent. Total oxygen dose was 184 AHs. Symptoms resolved in 4-6 weeks and patient received another 10 twice/day treatments. The child experienced the same neurological deterioration which again resolved in 2-3 weeks. Total oxygen dose was 201 AHs. Five-six weeks later the child received another 50 treatments. During this course his speech improved, but he became extremely fatigued/lethargic. Total oxygen dose was 289 AHs. A three-week break did not resolve the fatigue, so child received another 50 treatments. During this course he became extremely energetic, talkative, happy, stopped taking naps, and was "wired." Total oxygen dose was 377 AHs. Two to three days after returning home he developed extreme lethargy, loss of speech, pallor, and global deterioration in neurocognitive function. Three months later patient's mother demanded a decrease in pressure to 1.5 ATA and the child received 30 treatments. This was well tolerated and the child improved speech, balance, motor, and cognitive functions. Two months later the child received another 18 treatments, had further improvements, but on the last day developed severe muscle spasms in his good leg. Hospital Emergency Department evaluation revealed new clonus in multiple extremities. Total oxygen dose was 449 AHs. Metabolic blood workup was negative. In retrospect mom noticed that during some of his treatment courses he would develop anger and aggression along with the other symptoms noted above.
45. The patient was a two-year-old shaken baby boy with 30 myoclonic seizures/day whose seizure frequency increased to 50/d after his transcontinental plane flight to a hyperbaric facility 22 months after his injury. He received 38 bid 1.75/60 HBOTs. His seizure frequency decreased by the 4th HBOT and seizures were gone by the 38th HBOT. He was left with eye twitching that occurred a few times/day every other day, marginal improvement in head control, increased alertness, was more awake, resolution of fisting, more open hands, and developed grasp. Simultaneously, he tolerated a 60% reduction in Depakote dose. Two months later the child underwent another 40 1.75/60 HBOTs during which he experienced a rebound in seizure frequency to greater than his original pre-HBOT condition. His dose of oxygen was 137 AHs. Patient subsequently had additional rounds of HBOT upto a total of 150 HBOTs at unknown dose with no neurological improvement.
46. The patient is a 2-year-old boy with hypoxic ischemic encephalopathy and seizure disorder who began 1.25/60 bid 6d/wk (once on Saturdays) HBOTs. The pressure was increased in increments to 1.5 and finally 1.75 ATA as the child's seizure frequency decreased and changed from 400-500 short mild seizures/day to >100 prolonged severe seizures/day. By the 94th total treatment the severity of the seizures had increased and necessitated hospital admission for evaluation and control. In-hospital the child was placed on the ketogenic diet. Total dose of oxygen was 141 AHs. After one week of the ketogenic diet seizures decreased to 70/week. HBOT resumed at a lower dose (1.25/60) qd, 6d/wk for 35 more treatments. Seizures progressively decreased during and after the

HBOT and the child continued to improve neurologically. One year later the child began HBOT at 1.25/60 qd and bid for 30 treatments followed by a maintenance schedule 3-5x/wk intermittently to a total of over 200 HBOTs. At conclusion of HBOT seizures were occurring only on awakening or falling asleep or when the child was fatigued.

47. The patient was a 14 y.o. male with juvenile dermatomyositis (JDM). Initial signs and symptoms were extreme fatigue, pain and weakness of extremities with muscle aches, red/inflamed nail beds, and red raised rashes on eyelids. He was treated by numerous physicians for a host of ailments, including allergies and iron overload before being diagnosed with JDM. He was started on daily prednisone and symptoms worsened: could not dress himself, lift his head, and could barely walk. He was seen by a specialist who admitted him and resumed prednisone at increased dosage – 1000 mg/d for 1.5 years. He experienced emotional lability, extreme fatigue, "brain fog". While on prednisone he was treated with a variety of different medications, including IVIG. Upon weening of the prednisone, the side effects of the IVIG increased, especially excruciating HA unresponsive to medication. Patient was given a drug holiday and after 4-5 months without any medications his condition improved. The parents, both in the medical field, then tried IV infusions at home with a Meyer's Cocktail: Glutathione, B vitamins, C, Zinc, and amino acids. The parents sought HBOT at a nearby facility (6,000 ft. altitude) and received 9 HBOTs at 1.55 ATA with 100% for 60 mins. at depth. The patient experienced cognitive improvement but became increasingly irritable and anxious after each treatment and finally refused to go in the chamber. Total oxygen dose was 14 AHs. The anxiety and irritability receded over the next 3-4d. Patient's parents consulted the primary author and were evaluated in New Orleans three months post last HBOT with qEEG HBOT dosing. A dose of 1.3 ATA/45 mins. TDT with 4 liters oxygen by vented non-rebreather mask was delivered qd for 19 HBOTs over 24d. Patient experienced a generalized increase in energy, mood, strength, coordination, reduction in muscle pain, improved forearm rash/peri-cuticle erythema/peri-orbital erythema, decreased brain fog, absence of joint pain, and improved cognition. Repeat blood draw on return home revealed a reduction in JDM inflammatory markers.
48. The patient is a 39 year-old male with acoustic trauma from 140 Db exposure x 3h, right ear unprotected. Walked out of room and severe tinnitus right ear. Multiple episodes in past (musician) which resolved overnight. Left ear, questionably no symptoms, but tinnitus in right ear so loud couldn't tell if any tinnitus in left ear. Over next 16d slow improvement in right ear, no tinnitus in right ear. Saw ENT at day 16, audiogram normal bilaterally, put on Klonopin. Started HBOT day 17, 2.0 ATA/60 at depth bid x 4 HBOTs, then 2.5 ATA/60 at depth bid x 2 HBOTs, then 2.0 ATA/60 at depth bid x 4 HBOTs, total 10 HBOTs in 7d, multiplace, hood. Right ear tinnitus worsened in the injured ear and tinnitus erupted in the uninjured ear after the $9^{th}$ HBOT. No middle ear barotrauma with the HBOTs. Total oxygen dose was 19 AHs. Repeat visit to ENT, audiogram, new high frequency hearing loss left ear. No pain or vertigo. Intratympanic membrane steroid injection left ear. Four weeks post, calls author, tinnitus "same or worse both ears, going nuts."
49. The patient is a 12 year-old girl with severe TBI at 3.5 y.o. Significant neurological residual. Biomedical engineer father consulted primary author multiple times over a number of months regarding HBOT for his daughter and met with him at a scientific meeting. No further contact with father until two years later when father called to

attempt sale of his hyperbaric chamber to primary author. Unbeknownst to primary author father bought hardshell hyperbaric chamber from a physician, placed it in his home, and commenced treatment of his daughter 6.5 years after TBI for 420 HBOTs over two years. Dose was 1.68-1.82 ATA/unknown duration. Child experienced neurological improvement, but then developed grand mal seizures (unclear AHs, but seizure onset months before death). Father aware of literature on HBOT and oxygen toxicity as well as literature suggesting HBOT could benefit seizures. He continued to treat his daughter with HBOT, even rushing to place daughter in chamber during active seizure to "quell" the seizure. Reported that HBOT accelerated recovery of consciousness from post-ictal state and felt this was beneficial to seizure condition. Seizures intensified and became intractable as HBOT continued and child found dead in bed of presumptive seizure. Total oxygen dose was estimated at 735 AHs calculated from average 1.75/60 HBOT profile.

50. The patient is a 24 y.o. female, Type II decompression illness, brain and spinal cord, eight day delay to treatment with U.S. Navy Table 6, a U.S. Navy Table 5 daily for two days, and two U.S. Navy Table 9's/day for two days with some initial relief of dizziness, nausea, fatigue/exhaustion, problems walking, arthralgias on the entire right side of her body (worse in the arm than leg), paresthesias in the right hand, left hand and left foot, pain in the right arm, blurry vision, confusion, feeling "spaced out," and trouble thinking. Then developed progressive increase in dizziness, light-headedness, migratory paresthesias and pain, decreased balance, decreased vision, and severe headache during the last two HBOTs. Total oxygen dose was 27 AHs. HBOT was discontinued and these negative symptoms partially improved over the next 3d. In a conversation with a hyperbaric technician at a hyperbaric center in her home country she was informed that she would be paralyzed unless she received extensive HBOT. On the fourth day she took a 10h transcontinental flight home, developed right upper extremity pain in flight and felt sick and lethargic on landing. Four hours later she flew 1.5h to her home UK country and went to a hyperbaric center to receive a 2 ATA/60 min. HBOT. She received an additional daily 2ATA/60 x 3 HBOTs with some improvement then regression over the weekend. She then began 71 2.2 ATA/90 minute twice/day with 30-minute surface interval, 6d/week HBOTs. She had worsening of pain during the first 30 treatments then improvement in pain, but progressive slow improvement in her other symptoms until the last two weeks when all of her symptoms progressively worsened. She noted a total body buzzing/ paresthesias feeling in the chamber and then between treatments. On the last treatment she developed an abnormal sensation in her head, severe headache, removed the oxygen mask, and aborted the treatment. On chamber exit and over the next two days she was "non-functional," cognitively impaired, and extremely fatigued. Total oxygen dose was 185 AHs. She consulted the primary author who felt she was oxygen toxic and recommended immediate cessation of HBOT, tricyclic anti-depressants for the worsened pain, and a neurology consult and possible future HBOT at a much lower dose for residual DCI symptoms. Over the next month the patients oxygen toxicity symptoms resolved and she underwent three HBOTs at 1.25 ATA/60 mins. and experienced worsening of her symptoms. HBOT was discontinued.

Table 4: Chronic Oxygen Toxicity cases at <1.5 ATA oxygen

51. The patient is a 10 y.o. male with autism spectrum disorder at 2 y.o. who had had speech and occupational therapy, special education classes, biomedical interventions, dietary therapy (casein-free, gluten-free), and supplements with significant improvement. The patient was in mainstream school classes with other neuro-typical kids. Despite his higher level of function he had verbal and basic social skill deficits. He underwent 60 HBAs at 1.3 ATA/60 minutes with air in a portable hyperbaric chamber, 5/wk., over 3 months. The parents appreciated improvements and resumed HBA 4 months later at 1.3 ATA for 2 treatments then 1.5 ATA for 7 treatments, all for 60 minutes each daily. The patient's mother terminated treatments "immediately" after the 9$^{th}$ HBOT due to new onset of tics (involuntary movements in his wrists and legs) with head shaking. Total oxygen dose was 19 AHs. Two days later the mother contacted the author noting that the tics were still preset, but waning.

52. The patient was a 38 y.o female multi-trauma patient with a moderate TBI and extensive injury on MRI. Eleven months later she was treated with multiple rounds of HBOT: 1.5/60-90 x 20, two-week break, 1.5, 1.75, 2.0/90 bid x 5d and qd x 3, two-week break, 2.0/60-90 x 3 with improvement in headaches and cognition. She received additional HBOT at home in a portable chamber one month later at 1.3/90 bid, 10L O2 by venti-mask (95% O2) x 15 with further improvement in headaches, TBI, and cognitive symptoms. Patient was able to work. One month later patient started sleeping in the chamber for hours at a time at 1.3 ATA/2-3 hrs. with 10 liters/minute O2 (95% O2) by venti-mask, qd, 5d/wk x 15. After the 10$^{th}$ treatment (61 total) there was a return of severe migraines, all TBI symptoms worsened (Total oxygen dose to this 10$^{th}$ HBOT was 138 AHs). During the last few of the 15 HBOTs in that month she experienced increased head pressure and head burning sensation in the chamber. She continued home treatments in the portable chamber during the following month at 1.3/2-3 hrs with 10L O2 (95%) venti-mask, qd, 5d/wk x8. Symptoms severely worsened, including HA's, agitation, decreased vision, and sound sensitivity. Total oxygen dose was 156 AHs after the 67$^{th}$ HBOT (first in this block of 8 HBOTs). HBOT continued, but the patient decreased the treatment time for 4 qd HBOTs at 1.3/20 mins. with 10L O2 venti-mask, no change in symptoms, 3d break, then 2 HBOTs at 1.3/60 with O2 mask. Nine-day break ensued then she performed 2 treatments where she fell asleep in the chamber, 1.3/3h with 10L O2 by mask. There was a severe exacerbation of symptoms (Total oxygen dose was 189 AHs), followed by a two-day break, and travel from sea level to over 7,000 feet for medical treatment where she received 3 HBOTs at 1.5/90 and 1.75/90 on oxygen in a hardshell chamber. During treatment she had an abnormal sensation in her head with worsening of headaches and TBI symptoms. Total oxygen dose was 196 AHs. She was administered two daily doses of Narcan with some relief, then received stem cells, flew home, and experienced pressure in her head during flight. On landing she had had an exacerbation of her symptoms that persisted the rest of the month. Her physician started her on gabapentin with partial relief. The patient received two more HBOTs at home 4 months after her last HBOT at 1.3 ATA/95 mins, two-day break, then 1.3/45 with 10L oxygen by venti-mask. Her migraine headaches and other symptoms worsened. Total oxygen dose was 201AHs. The patient consults the primary author who diagnosed oxygen toxicity and recommended anti-oxidant treatments which were performed with partial transient relief. Sinequan was also recommended. Very limited HBA was recommended to test for anti-

inflammatory contribution at 1.15 ATA/25 mins. Partial relief of symptoms resulted. Two days later a repeat treatment was delivered, but the patient increased treatment time to 35 mins. Migraine headaches resumed 6h later, followed by chest tightness the next day and sensation of inability to take a deep breath. Total oxygen dose was 201.2 AHs. HBA was stopped, chest symptoms resolved in 2d, however, migraines worsened.

53. The patient is an 18 y.o. female diagnosed with PPCS stemming from a concussion with LOC while entertaining in a bubble body ball at a basketball game. She developed a persistent postconcussion syndrome and underwent daily HBOT at 1.5 ATA x 1 and 1.7 ATA x 11 with 24.5% O2, all for 90-120 mins total dive time. She experienced increased agitation with the increasing number of HBOTs. After a 3d break she transitioned to a portable chamber at 1.3 ATA for 60 mins bid plus 1.3 ATA 90-120 bid, 7 d/wk with 10 L O2 by mask for 35 treatments. Because of symptom improvement she was instructed by the chamber rental person after a 12d break to increase treatment time in the chamber to 120-180 mins/treatment and to start sleeping in the chamber. Ten days later she resumed bid HBOTs, 7d/wk at 1.3 ATA/ 120-180 mins. with 10L O2/min by mask for 26 HBOTs and also began red light therapy to her head. By the $12^{th}$ – $13^{th}$ treatment she experienced worsening of her PPCS symptoms, but continued the HBOT and red light therapy. Total oxygen dose was 122 AHs. After the last of these 26 HBOTs she took a one-day break, decreased the dose to 60 minute treatments for 3 HBOTs with 10L O2, and continued her red light therapy daily. The patient felt better after the first two HBOTs, but after the $3^{rd}$ HBOT experienced increased fatigue and amount of sleep and discontinued the HBOT. Total oxygen dose was 169 AHs. Over the next 3 weeks the extreme fatigue improved, excessive sleeping resolved, and PPCS symptoms improved. She was evaluated in New Orleans by the author three weeks after her last HBOT and resumed HBOT at a much lower dose for residual PPCS symptoms and fatigue (38 treatments, 1.3 ATA for 45 mins, qd, 5d/week, ambient oxygen with air by non-rebreather mask). The patient experienced PPCS and fatigue symptom improvement through 28 HBOTS then had fluctuating symptom response in the midst of increased physical activity and a URI/sinus infection over the next 10 HBOTs. Patient experienced exhaustion and exacerbation of PPCS symptoms after the $38^{th}$ HBOT at which time HBOT was discontinued. Total oxygen dose was 175 AHs. Over the next 12 months the patient's symptoms improved and she was re-evaluated for remaining PPCS symptoms using qEEG-assisted HBOT dose-finding one year after the $38^{th}$ HBOT. Patient resumed HBOT at the same dose, 1.3/45 mins. with steady improvement in symptoms through 13 HBOTs. Patient finished with the least symptomatology since her accident, but still had mild residual symptoms. Over the next 12 months patient made no significant improvement. Activity was limited and she had not returned to school. She returned for additional HBOT at this time before attempting a return to school. She received 7 HBA's at 1.3 ATA/45 mins. and 1 HBOT at 1.3/45 with 4L oxygen by venti-mask and felt an improvement in mood, energy level, and activity. Due to her responsiveness at this dose she was subsequently transitioned to a portable chamber at home where the author helped treat her over the ensuing two years with progressive improvement while returning to school.
54. The patient was a 66 y.o. male who presented with a complaint of low back pain, cognitive slowing, anxiety to car racing and decreased performance. He was treated with

311 HBOTs over 12 years in hardshell and softshell hyperbaric chambers with occasional blurry vision on exiting the hyperbaric chamber. These episodes resolved overnight or by 24 hours. Treatment consisted of initial rounds of HBOT at 1.4 ATA/60 mins. with a single air break of 100% O2, for a total of 205 treatments with symptomatic improvement. The patient then transitioned to HBOT in a portable chamber with sustained improvement at 1.3 ATA/50 with 10 liters/minute 95% O2 by NRB mask for a total of 106 treatments at home under the direction of the author. One month after the 311th HBOT he fractured his patella and underwent surgical repair 6d later. He was unable to get into the portable chamber, so he was instructed to start using NBO with 95% O2, 10L/min by NRBM for 45 mins 4d after surgery and 10d after the fracture. The following day he underwent the 2nd NBO treatment and developed blurry vision, brain fog, and could not focus his thoughts. Total oxygen dose was 397 AHs. His vision and brain fog resolved overnight. The following day another NBO treatment was attempted. Twenty minutes into the treatment he experienced identical symptoms that lasted 3 days. An eye exam conducted in April 2022, showed he had bilateral cataracts. A previous exam in January 2021 showed no cataracts.

55. The patient was a 13 y.o. girl with a febrile illness, viral encephalitis, status epilepticus, pentobarbital coma x 46d, acute care hospitalization + rehabilitation hospital for 4.5 months who went home for 2.5 months, was re-admitted to a 2nd rehabilitation hospital for 4 months, and heavily medicated. At the parents' demand the meds were weaned and she was discharged home. Seizures recurred 4 months later with pre-menstruation. She was verbally communicative only when in water. Main symptoms of aggression, lost toilet-training, no social interaction, sleep disruption, no relationship with mother, seizures (last seizure one month pre-evaluation), no awareness of danger (housebound). HBOT, 1.2 ATA/60, bid, 5d/week x 32 treatments with improvement in aggression, toilet-training, conversation, relationship with mother, sleep. One small pre-menstrual seizure first week of HBOT. From the 33rd-35th HBOT she regressed with significant increase in aggression/ violence and loss of other gains. (Total oxygen dose was 40 AHs.) Dose was decreased on the 36th HBOT to 1.1 ATA/60 mins. through 41st HBOT and changed to qod frequency with reversal of regression, spontaneous use of bathroom, no aggression, improvement in behavior, and no seizures in 3 months. Further improvements on two-month break, documented by multiple therapists and physicians, then resumes HBOT at 1.1 ATA/60 mins. x 37 treatments in 2 months. Improvement through 22 HBOTs despite 3 seizures, then regression of improvements with increasing intermittent aggression. Total oxygen dose was 74 AHs. Treatment stopped at 37 treatments due to escalating aggression, decreased sleep, agitation. In the week following, regression of deterioration. Over next 5 months patient progressed to in-home schooling 3-5h/day and eventually normal school 1.5-2h/day, 2 petit mal seizures, and aggression only at pre-menstruation. Resumes HBOT 9.5 months after last treatment, same dose, 4 HBOTs/11d, improvement, 3 HBOTs one year later, then 2 HBOTs/month every other month for 6 HBOTs. Patient experiences burst in cognition and improved behavior with each short course of HBOT. Attending self-contained classroom (patient is the only student) at mainstream school, 4h/d, 5d/ week. Residual problems with impulsivity, dealing with new experiences, socialization, sensitivity of scalp, urinary incontinence, and rare stool incontinence.

56. The patient was a 25 y.o. male, 16 months post motorcycle accident with severe TBI, SDH, SAH and ICPs to 60. Subsequent hydrocephalus, shunt placement, shunt revision.

Acute care and rehabilitation hospitalization of 5 months. At time of evaluation, ambulatory, one-two occasional words, but mostly non-communicative, follows some commands, bradykinesia, poor balance, incontinent of bowel and bladder, no appetite, poor visual processing, marked cognitive deficits, decreased motor function, but excessive force with motor movements/grip/hug. HBOT, 1.4ATA/50, qd, 5d/week x 39 with improved balance, reading some words, feeding self 100% of the time, mild improvement in motor function/bradykinesia, affect, mood, energy level/motivation, more affectionate, more relaxed, cognitively improved with more speech (increased volume, sentences). One month break, further improvement in speech, mobility, spontaneous activity, singing more, less aggressive grip/hug. Resume HBOT, same dose, mother notes using bathroom independently and on cue, increase in conversation, spontaneous reading, improved balance, answering some simple questions by 49th HBOT. By 56th HBOT 2d regression of behavior with increased agitation, confusion, cognitive decline (putting whole egg with shell in frying pan, throwing utensils in the trash), elopement, slamming doors. Total oxygen dose was 65 AHs. Dose of HBOT decreased to 1.15 ATA/45 mins. By 61st HBOT further improvement with regression of abnormal behaviors, more normal facial expression/less confusion. 63rd HBOT, using bathroom spontaneously for bowel movements. 68th HBOT further increase in speech/conversation. 69-74th HBOT increasing agitation in the chamber: rubbing head vigorously, yelling. Simultaneously, disrupted sleep, loud voice, vigorously rubbing skin, bouncing legs. HBOT discontinued. Total oxygen dose was 76 AHs. Over next 17d regression of untoward symptoms/agitation. Net gain of improved balance, motor function, reading individual words, appetite, feeding self, energy level, spontaneous activity, fine motor function (guitar-playing, utensils/finger-feeding), mood, ADLs, cognition.

One month later mother takes patient for "high performance neurofeedback" and transcranial magnetic stimulation for 4-5 months. Patient experiences return of aggressive behavior, agitation, "hyped up," not sleeping, walking on tiptoes. Over next 9 years mother tries multiple therapies to reverse deteriorated condition. Some response to laser brain therapy and integrated listening therapy, but exacerbation with multiple medications. One and one-half years post last HBOT mother buys home chamber and does 24 HBAs with no effect. Returns for HBOT 8.5 years post last HBOT with primary author to attempt re-dosing. On exam very agitated, pacing, not following commands, aggressive, clutching, baring teeth, shaking examiner and other clinic staff. HBOT resumed at last dose used 8.5 years ago, 1.15 oxygen/45 minutes. Patient became increasingly agitated in chamber. Toward end of chamber was grimacing, baring his teeth, rubbing his head vigorously, very agitated. Exited chamber and was angry, aggressive, agitated. Poor sleep that night. Agitation continued the next day and attempted lower dose at 1.3 ATA/45 with 4 liters/minute oxygen by mask. Wouldn't wear mask. Given straight air treatment. Calmer post treatment. Over next 9 days received 6 HBAs at 1.3/45 mins. with progressive calming, improvement in behavior, mood beyond pre-HBOT level, started answering questions with one-word answers. Patient no longer clutching/shaking people, more appropriate social interaction. Patient discharged to resume additional low dose treatment under author's guidance at home.

57. The patient is a 2.5 year old boy, 3 months post-drowning with cardiac arrest. Early seizures in the hospital. Ventilator x 3 weeks, tracheostomy. Discharged home 56d post

admit. Intermittent normobaric oxygen begun bid 10 weeks post-accident, 11d before HBOT for 8d with progressive improvement in awakeness, eye focus, some visual tracking of sounds and objects, head and body movement, head control, tone, and sleep, but developed spasms/arching of back. HBOT begun with HBA at 1.3/45 mins qd, 5d/week. Improved focus, storming, tone, alertness through 20 HBAs. Dose increased to pure oxygen at 1.15 ATA/45 for 7 treatments. Increased arching in chamber on 1st treatment then reductions in tone and storming. After 7th HBOT (#27) had episode of diaphoresis, increased arching with myoclonus (1st ever), no sleep in 48h, no infectious symptoms. Total oxygen dose was at 12 AHs. HBOT stopped. Negative symptoms ceased over next 5d, dose changed back to HBA, tolerated 1st HBA. After 2nd HBA increased storming, no sleep for 72h. Total oxygen dose was 12.4 AHs. Cessation of negative symptoms by 5th day. One additional HBA at lower dose delivered 1.15 ATA/45 mins. Patient developed storming and spasm/arching again. HBOT was discontinued. Total oxygen dose was 12.6 AHs. Severe symptoms abate over the next 5d, but the patient is left with tone/arching at pre-HBOT level. Other gains maintained as well as decreased vomiting, secretions, suctioning. Due to positive gains despite what appeared to be extreme sensitivity to even HBA dose family leases portable chamber for home and begins treatment over one month after last HBA at 1.15 ATA air/50 mins. 1st HBA was tolerated. After 2nd treatment had a recurrence of the extreme agitation, inability to sleep, and heart rate to 170s-180s. Total oxygen dose was 13 AHs. Treatment continued for a total of 12 HBAs and was stopped by parents due to continued agitation, prolonged awakefulness, and elevated heart rate.

58. The patient is a 22 y.o. female with a prior history of Lyme Disease, auto-immune encephalopathy/meningitis vaccine reaction, secondary OCD, mild TBI, and electrographic seizure disorder who presented 2.5 months post herpes encephalitis with fatigue, headaches, cognitive impairment, diffuse body pain, and heat intolerance. Patient received 40 HBOTs at 1.15 ATA/45 mins. qd, 5d/wk in eight weeks, experiencing symptomatic improvement. Over the subsequent year the patient experienced an initial clinical deterioration with dyskinetic movements, fatigue, vomiting, requiring TPN, and was found to have elevated intracranial pressure and a positive tilt table test. She improved symptomatically with medication and presented for additional HBOT 15 months post last HBOT with the original constellation of symptoms and now dyskinetic movements. On evaluation the patient was improved over her evaluation pre-HBOT 17 months previously. HBOT resumed at the previous dose and 10 minutes into the first treatment patient experienced lip twitching, jaw spasm with trismus, and gross arm tremors. Chamber gas was immediately switched from oxygen to air and the symptoms resolved in 7 minutes. Total oxygen dose was 35 AHs. The patient completed a 45 minute treatment and three subsequent qd HBAs at 1.15 ATA/45 mins. with some improvement in her baseline symptom complex. HBA dose was changed to 1.3 ATA/50 mins., qd, 5d/wk. x 9 treatments with progressive symptomatic improvement.
59. The patient is a an 83 y. o. female who had a stroke during general anesthesia/surgery for laryngeal implant at 68 y.o. Persistent dizziness and short-term memory loss were present immediately post-surgery and became permanent. Eleven years after the stroke, HBOT was initiated at 1.5 ATA/60 mins. for 21 HBOTs. There was an improvement in cognitive symptoms and dizziness. HBOT was stopped after the patient contracted an upper respiratory infection and returned home. During the subsequent four years the

patient continued to cognitively decline, was unable to drive, had to be transitioned from independent to assisted living, required assisted ambulation for intractable arthritis and low back pain/sciatica, and was diagnosed with dementia. HBOT was resumed by her son, a general surgeon, at 1.3 ATA/60 mins qd, 5d/wk, with 10L 95% O2 by non-rebreather mask in a portable chamber. Clinical improvement ensued. The patient was able to discontinue both anti-dementia drugs, two antidepressants, non-steroidal anti-inflammatories, narcotic pain medicine, and achieved independent ambulation. By treatment 135 she developed increased agitation, irritability, paranoia, combativeness, and anger. Total oxygen dose was 198 AHs HBOT was stopped. Negative symptoms waned over one month and HBOT resumed with periodic breaks to 250 total treatments. The patient experienced progressive improvement in cognition, social interaction, and quality of life. HBOT was discontinued at the patient's request with residual cognitive impairment, but she lived another 5.5 years medication-free with mild decline in cognition until sustaining a fracture/dislocation of the shoulder from a trip/fall. The patient refused surgery, expressed that it was time to move on and died peacefully in two weeks.

Table 5. Withdrawal syndrome.

60. The patient is a young girl with dystonia who underwent forty 1.75/60 - bid HBOTs and experienced a marked improvement in dystonia. One month break ensued in which the patient completely regressed. An additional 20 1.75/60 bid HBOTs were administered and again the patient improved but regressed upon cessation. After approximately a one-month break the mother stated she was more determined to ensure durability of the hyperbaric-induced improvements and obtained 90 1.75/60 bid, 7 days/wk HBOTs. During this time the patient became "very energetic, active, healthy, and relaxed with improvement in dystonia again." Total oxygen dose was 263 AHs. A three-week break followed and during the second week the patient had a dramatic reversal where she became extremely rigid, twisted, and "her legs are turning, her pelvis is deviated, her left hip ... she is not eating well." Child was in state of constant involuntary movement "in all directions" of "her body, legs, arms," grinding her teeth. Mother described the patient's conditions as the worst she had ever been. Mother then consulted a hyperbaric Internet chat group and became aware of author's recommendations for lower pressure HBOT. She obtained and installed a hardshell chamber in her home and gave her daughter a lower pressure dose of HBOT (1.4 ATA/60, qd, 5d/week x 20 HBOTs). The child experienced partial relief, but still had high tone. The HBOT was followed by six weeks of physical therapy and the patient had slight additional improvement. She then delivered additional HBOT at 1.27 ATA/60 qd, 5 days/wk for 7 treatments combined with physical therapy. The patient experienced improvement with reduction in tone, dystonia, increased appetite, strength, and improvement of the negative effects described during the withdrawal period above (pelvic twisting). Total oxygen dose was 263 AHs.
