# Supplementary material for: Acute and chronic central nervous system oxidative stress/toxicity during hyperbaric oxygen treatment of subacute and chronic neurological conditions
Source: Front Neurol. 2024 Mar 4;15:1341562. doi: 10.3389/fneur.2024.1341562 (PMC10946424; doi:10.3389/fneur.2024.1341562)
Supplement: Supplementary file 3 [file Table_1.pdf]

SUPPLEMENTARY TABLES S1-S5: Data Tables 1-5

Table 1. Acute Oxygen Toxicity (OT). Cases experiencing OT in ≤ 5 HBOTs. **BOLD:** cases treated at outside facilities. Subject # corresponds to subject #'s in Supplementary File S2. HBOT schedule: x ATA/y = pressure level in atmospheres absolute/minutes of total treatment time unless specifically designated. qd=once/day, bid=twice/day.

Sub-ject	Age/Sex	Diagnosis	Time from injury to HBOT	HBOT Schedule	Num-ber of HBOTs at Symp-tom Onset	Signs/Symptoms of Ox Tox	Cumulative HBOT dose (ATA-hrs.)
**1**	32,F	Decompression Illness	30d	"Standard Navy decompression tables," 2.0 ATA, 75% FiO2/120, qd x 3, 2.0 ATA, 75% FiO2/60 x 24	1	Nausea, vomiting, weakness, pallor, diaphoresis	3.0
**2**	Young Child,M	Seizure Disorder	years	1.75 ATA/60, bid, 5d/week	5	Increased seizure frequency	8.7
**3**	2,M	Drowning, spastic quad cerebral palsy (CP)	5mos.	1.75 ATA x 1, 2.4 ATA x 1, 1.75 ATA x 17, 2.0 ATA x 1, all 60 mins.	2	Uncontrollable shaking arms, legs, chin, clonus in chamber	4.1
**4**	44,M	Anoxic Brain Injury, seizures	4y	1.5/45 x 1, 1.75/60 x 1 and 1.75/120 x 1 bid, 1.75/60 x 4 bid, 1.5/45 x 2, 1.5/60 x 1	2	Incoherent, couldn't eat after 2 HBOTS, "under the influence" after 3rd HBOT, lost balance, gait,	2.9

						swallow , and all neuro gains of 2 previous years after 7th HBOT	
**5**	15,M	Rasmussen's encephalitis (seizures)	10y	1.7/90 x 18 bid, 1.5/60 x 5, qd, all 6d/week	1	Seizures 20 mins. into each HBOT, increasing seizure frequency with HBOT, status epilepticus, hemispherectomy	0.43
**6**	Adult M	Amyotrophic Lateral Sclerosis, TBI	mos.	1.75/60 qd x 3, 2.0/60 x 1	4	Deterioration with drooling, decreased speech	7.25
**7**	95,M	Stroke; stroke	2wks; same day	(1.35 ATA/60, qd, 6d/wk., x 3000), 1.5/60, 4h Surface interval, 1.1/60 (abort at 20 minutes); next day 1.1/60 (abort at 20 minutes).	2	No signs/symptoms from 3000 HBOTs, Intense head pain and sensation of "head squeezed" followed by neurologic deterioration. Next day confusion, and somnolence, but conversant, grand mal seizure during HBOT that day. 15 more seizures until death the following afternoon	4052;<br>1.7 (4054)

Table 2. Chronic CNS Oxygen Toxicity (OT): Cases experiencing OT at 1.5 ATA oxygen. **BOLD:** cases treated at outside facilities. * = cases treated at outside facility and by author. Subject # corresponds to subject #'s in Supplementary File S2. HBOT schedule: x ATA/y = pressure level in atmospheres absolute/minutes of total treatment time unless specifically designated. qd=once/day, bid=twice/day

Subject	Age/Sex	Diagnosis	Time from injury to HBOT	HBOT Schedule	Number of HBOTs at Symptom Onset	# of consecutive HBOTs at Symptom Onset	Signs/Symptoms of Ox Tox	Cumulative HBOT dose (ATA-hrs.)
8	46,M	Cerebral DCI, dementia	7mos.	2.0, 2.4, 1.5/90 qd x 31; 1.5/90,1.5/60 bid and qd 1.5/60, 7d/wk. x 79	31;110	31;79	Dizziness, restlessness, agitation; dizziness, restlessness, agitation, extreme anxiety in chamber	91;215
9	4,M	Cerebral palsy	4y	1.5/90 qd-bid, 5d/wk x 103, 1.5/90 qd-bid, 5d/wk x 91	185	82	Regression: decreased tone and fine motor, tremor, startle, hyperactivity, drooling	416
**10**	19,M	Ischemic/hypoxic, sepsis	3.8y	1.5/60 bid x 40, x 40, 1.5/90 qd x 40, x 40 ; 1.5/90 qd x 15; 1.5/60 qd x 6	135;164;181	15;4;6	behavioral deterioration, offensive, hyperactive, rambunctious, aggressive; behavioral deterioration, hyperactive, demanding; fidgety, hyperactive, short-tempered	244;309; 349
11	46,M	Carbon Monoxide	6mos.	1.5/90 x 40, 1.5/90 x 30, qd, 6d/wk	65-70	25-30	Dizziness, dysphoria, visual symptoms, nausea	146
12	50,M	Carbon Monoxide	6mos.	1.5/90 x 40, 1.5/90 x 30, qd, 6d/wk	65-70	25-30	Dizziness, dysphoria, visual symptoms, nausea	146
13	33,M	Carbon Monoxide	6mos.	1.5/90 x 40, 1.5/90 x 30, qd, 6d/wk	65-70	25-30	Dizziness, dysphoria, visual symptoms, nausea	146
14	52,M	Carbon Monoxide	2.5y	1.5/90, bid, 6d/wk x 40, then x 33, 1.5/60 bid x 2	75	2	Internal jitteriness/"racing", anxiety, "nervousness" in chamber	167
15	60,M	Stroke; TBI with brain hemorrhage	2.5y;3 mos.	1.5/90, qd-bid,5d/wk x 40, 1.5/90, qd-bid, 5d/wk x 35	75	35	increase in activity/energy level, compulsive reading, decreased sleep, euphoria, irritability, short-temper, anger, arguing, aggression, agitation, and irrational thinking/behavior	169

**16**	33,M	Severe TBI	3y	1.5/60, qd, 5d/wk x 110	110	30	Agitation, aggression, bizarre, and uncontrollable behavior	165
**17***	4,M	Drowning	1y3m os.	1.5/90, bid, 5d/wk x 80; 1.5/60 bid x 34; 1.5/60 bid x 31; 1.5/60 bid x 35; 1.5/60 bid x 10	20-25; 112;144;177; 190	20-25; 32; 30; 32; 10	Frequent myoclonus and fatigue; same; same; same plus seizure; extreme irritability, verbal outbursts, aggression	45; 228; 276; 326; 346
**18**	19,M	Drowning	7wks; 1y7m os.	2.0/90 x 30 bid, 1.5-2.0/30-60 x 190 bid, 1.5/90 qd x >281	276; > 501	56; >224	Boundless energy; Increasing uncontrollable behavior: aggression, anger, and irrational thinking, requiring institutionalization	465; 971
19	1y6m os.,F	Quad CP, seizures	1y6m os.	1.5/60 x40, bid, 1.5/60 qd-bid x 37, 1.5/60 qd x 13,11,12,13,6; same x 7, same x 1; 1.25/60 qd x 6	132;140;146	6;1;6	Became "wild" with extreme exacerbation of tone, extensor posturing, and decreased appetite; hyperactivity, increased tone, sleep disruption; twitching in chamber; "wired," hyperactive, increased tone, decreased sleep; twitching in chamber	197; 210; 217
20	3,F	Athetoid CP	3y	1.5/60 x 42, x 38, x 12, 10, 10, 10, 10, 3, all bid	135	3	Jittery/tremulous in chamber	203
21	10,F	Quad CP	10y	1.5/60 x 40, x 25, all bid	65	65	Lethargy, startling to loud noises, and began biting individuals. Diffuse hyperreflexia and clonus on exam.	97
**22***	3,M	Spastic diplegic CP, subcortical seizure disorder	3y	1.5/60 x 37; 1.5/60 qd-bid x 42; 1.75/? x 1; 1.3/55 qd x 8	36; 80; 88	36; 1; 8	Increased myoclonus frequency; Grand mal seizure in chamber; Complex partial seizure in chamber	54; 120; 129
**23**	8,F	Severe TBI, persistent vegetative state	3y, 2mos.	1.5(?)/60(?) x 75, bid; 1.5(?)/60(?) x 30, bid; 1.5/? x 15, qd, 1.5 then 1.3/60(?) qd, x 5-15 (?) q 2-3 months	40; 105; At least 120	40; 30; >15	Increasing irritation; increasing agitation/irritation; return of seizures, status epilepticus, cardiac arrest, death	Estimated 60; 158; >180

24	9,M	Autism	7y	1.5/60 x 40 qd, 1.5/60 x 25	61	21	Mood swings, speech, agitation, hyperactivity, recurrent autistic obsessions/behaviors	91
**25**	Elderly,M	Creutzfeldt Jakob Disease	Unkn.	1.5/unknown, qd-bid, (estimated at least standard 60 minute treatments)	At least 10	At least 10	Increased restlessness, spasticity, aggressiveness, incontinence	Estimated at ≥ 15
**26**	2y11 mos,F	Birth injury, Seizure at birth, Developmental Delays,	2y, 6mos.	1.5/60 x17, 1.5/60 at depth x 15	32	15	Jerkiness (in chamber), complex partial seizure during 32nd HBOT	48
**27**	7 mos., F	Herpes meningitis, encephalitis	1 mo.	1.15/45, qd, x 11, 1.5/90 bid at depth qd x 5	13; 15; 16	13; 2; 1	Seizure at depth 13th, 15th, and 16th HBOT	12.5; 15.5; 16.3
28	50,F	TBI, PPCS	16mos.	1.5/60 x 39, qd, 5d/wk	39	39	Fatigue with associated confusion	59
29	49,F	TBI, PPCS	8y	1.5/60 x 30 qd, 5d/wk	22	22	Euphoria followed by extreme fatigue, cognitive reversal and paresthesias with additional treatments	33
30	45,F	TBI, PPCS	3.5y	1.5/60 x 37, 5d/wk	36	36	Fatigue by 32nd HBOT, confused and unable to converse by 36th HBOT	54
31	48,F	TBI, PCCS	22mos.	1.5/60 x 37, 5d/wk	37	37	Increasing fatigue	55
32	38,M	TBI, PPCS	1y	1.5/60 x 34 qd, 5d/wk	29	29	Twitching, sleep disruption, dizziness, fatigue, weakness, shakiness, blurry vision, nausea, flu-like symptoms	43
33	45,M	TBI x 3, PPCS	12y; 8y; 10 mos.	1.5/60 x 40 qd, 5d/wk	38	38	Increasing fatigue, “foggy-headedness”	57

**34**	3F,	CP, spastic diplegia	3y	1.5/60 x 40 bid	21	21	Decreased spasticity, then progressive regression to ~baseline tone (HBOT 20-40)	31
**35**	15,M	Birth injury, ADHD, autism, TBI/PPCS	8 mos. post TBI	1.3/90 x2, 1.5/90 x25, 1.3-1.75 (average 1.5)/90 x 29; 1.5/45 x 9	47;63	20; 9	Increasing fatigue, agitation, frustration, HA in chamber, dizziness on chamber exit, difficulty processing information, absent-mindedness, “I don’t feel like myself;”	Estimated 105; 135, both based on average 1.5/60
**36**	51,F	COVID Long-Hauler’s Syndrome	6 mos; 2y	3 HBOT (unknown dose); 1.3/60 x 1, 1.5/60 x 8 qd	12	9	Extreme light-headedness, tachycardia, diaphoresis, near LOC, feeling of doom, “felt like going to die”	> 13
37	52,M	DCS Type II, brain & spinal cord	3y, 7 mos.	USN6AModified x 1, USN6 x 14, USN5 x 1, 3.5 years earlier, 1.5/90 bid x 72	62	62	Increasing paresthesias, dysesthesias in perineum, lower extremities, worsened autonomic symptoms	139
**38**	Elderly,M	Stroke	2y	1.6 (94% O2)/67.5 (from depth to surface) x 18, qd, 5d/wk	11	11	Aggressive, hyperactive; by 18th unable to sleep, very agitated, global deterioration on repeat qEEG	19

Table 3: Chronic CNS Oxygen Toxicity (OT): cases experiencing OT at > 1.5 ATA oxygen. **BOLD:** cases treated at outside facilities. Subject # corresponds to subject #’s in Supplementary File S2. HBOT schedule: x ATA/y = pressure level in atmospheres absolute/minutes of total treatment time unless specifically designated. qd=once/day, bid=twice/day.

Sub-ject	Age/Sex	Diagnosis	Time from injury	HBOT Schedule	Num-ber of HBOTs at	# of consec-utive HBOTs	Signs/Symptoms of Ox Tox	Cumu-lative HBOT dose

			to HBOT		Symp-tom Onset	at Symp-tom Onset		(ATA-hrs.)
**39**	58,M	Stroke/cerebral aneurysm rupture	3y	1.75 ATA x 3, 2.0 ATA x 5, 2.5 ATA x 11, 2.8 ATA x 1, 2.5 ATA x 5, all 60 mins., bid, 7d/wk; 1.75/60, bid, 7d/wk x 35	20; 60	20;35	Wild, combative, confused, incoherent; aggressive, angry, fatigued/lethargic	46; 119
**40**	21,M	Severe TBI, brain abscess, seizures	7.7y	1.5/60 x 8, 1.75/60 x 31, bid, 6d/week	18	18	Lost balance, gait, irritability, impulsivity, anger, aggression. Agitation in chamber	29
41	33,M	Cerebral DCI	1mo.	USN6Mono x 3, 1.5/90 x 40, bid,1.75/90 x 40 bid, all 80 7d/wk; 2.0/90 bid-tid x 15	83; 98	80; 15	Mildly irrational, hyperactive and euphoric; dysphoria, dizziness, tremor in chamber	211; 256
**42**	9,F	Drowning	5y	1.5/60 x 51, bid, 5d/wk, 1.5/60, qd, 6d/wk x 29, 2.0/120 x 19, qd; 1.5/60 qd x 25, 1.5/90 bid, 6d/wk x 24	99; 126	99; 27	Seized at depth; increasing fatigue, anxiety, hyperactivity. Also, discomfort in chamber.	196; 238
**43**	4,M	Diplegic CP	4y	1.75 (95%)/60 x 22, 1.75 (100%)/60 x 56, all at 11 HBOTs/wk	78	56	Seized at depth	135
**44**	12,M	Quad CP	12y	1.75/60 x 40, 1.75/60 x 10, 1.75/60 x 20, 1.75/60 x 20, 1.75/60 x 15; 1.75/60 x 10; 1.75/60 x 50; 1.75/60 x 50; 1.5/60 x 30, 1.5/60 x18. All HBOTs bid, 5d/wk.	105;115;165; 215;263	15;10;50;50;18	Speechlessness, decreased attention, cognition, balance, coordination, pallor, increasing lethargy, and was “rubbery legged, droopy and drunk”, falling, and became wheelchair dependent; same recurrent symptoms; fatigue/lethargy; hyperactivity, decreased sleep, “wired;” lower extremity muscle spasms, clonus in multiple extremities.	184;201; 289;377; 449

**45**	2,M	Shaken baby, myoclonic seizures	22mos.	1.75/60 x 38 bid; 1.75/60 x 40, bid	78	40	Increased myoclonic seizure frequency above baseline (>50/d)	137
**46**	2,M	Hypoxic ischemic encephalopathy, seizure disorder	2y	1.25-1.75/60 x 94, bid, 6d/wk.	94	94	Increased seizure severity necessitating hospital admission for evaluation and control	141 (calculated at average 1.5 ATA)
**47**	14,M	Juvenile dermatomyositis	2y8mos	1.55/60 x9, qd, at altitude	9	9	Increased irritability and anxiety	14
**48**	39,M	Acoustic trauma, tinnitus	17 days	2.0/60 x 4 bid, 2.5/60 x 2 bid, 2.0/60 x 4 bid	9	9	Tinnitus worse in injured ear, new tinnitus opposite ear with high frequency hearing loss. Four weeks post, tinnitus same or worse both ears.	19
**49**	12,F	Severe TBI	6.5y	1.68-1.82/at least 60 mins., qd, 5d/week	420	420	Initial neurological improvement, but then developed grand mal seizures which progressively worsened months before death, presumptively from seizures.	735 (calculated at 1.75 ATA/60 average)
**50**	24,F	Type II decompression Illness	8d	USN6 x 1, USN5 x 2, qd, USN9 x 2, bid, 2.0/60 x 3, qd, 2.2/90 bid, 6d/wk. x 71/2 months, 30 min. surface interval between HBOTs.	6;55	6;46	Increase in dizziness, light-headedness, migratory paresthesias and pain, decreased balance, decreased vision, and severe headache; total body "buzzing"/paresthesias in chamber and between HBOTs. During 81st HBOT severe headache, abnormal sensation in head, aborted treatment, cognitive worsening, fatigue.	27;185

Table 4: Chronic CNS Oxygen Toxicity (OT): cases experiencing OT at < 1.5 ATA oxygen. **BOLD:** cases treated at outside facilities. Subject # corresponds to subject #'s in Supplementary File S2. HBOT schedule: x ATA/y = pressure level in atmospheres absolute/minutes of total treatment time unless specifically designated. qd=once/day, bid=twice/day. NBO = normobaric oxygen.

Sub-ject	Age/Sex	Diagnosis	Time from injury to HBOT	HBOT Schedule	Number of HBOTs/HBAs at Symptom Onset	# of consecutive HBOTs at Symptom Onset	Signs/Symptoms of Ox Tox	Cumu-lative HBOT dose (ATA-hrs.)
**51**	10M	Autism spectrum with verbal and basic social skills deficits	8y	1.3air/60 x 60, qd; 1.3air/60 x 2, 1.5air/60 x 7, qd	69	9	New onset tics (wrist and legs), head shaking immediately after 9th HBA	19
**52**	38F	TBI	11 mos.	1.5/60-90 x 20, 1.5-2.0/90 bid x 10 and qd x 3, 2.0/60-90 x 3, 1.3/90 x15, bid 10L(95% O_2) venti-mask, 1.3/2-3 hrs x 15 qd, 10L(95% O_2) venti-mask; repeat identical last dose x 8 (see	61; 67	10; 1	Loss of initial improvement with return of severe migraines and worsened TBI symptoms, head pressure/burning in the chamber; worsening of all symptoms and decreased vision and increased sound sensitivity	135; 154

				history for additional details and more toxicity)				
**53**	18F	TBI	4 mos.	1.5 x1, 1.7/90-120, 24.5% O2, qd x 11, 1.3/60 then 1.3/90-120, bid,7d/wk x 35, 95% O2, 1.3/120-180, bid, 7d/wk., 95% O2 x 26; 1.3/60 95% O2 x 3; 1.3 air/45 qd x 38.	59; 76; 114	12; 3; 38	Worsening of PPCS symptoms: headache, fatigue, sleepiness, nausea, cognitive symptoms; increased fatigue and sleepiness; exhaustion and exacerbation of PPCS symptoms	122; 169; 175
54	66M	cognitive decline, anxiety	5 years	1.4/60 x 205; 1.3/50 x 106, 95% O_2 NRBM; NBO 10L (95% O_2) NRBM for 45 min x 2.	311/12 years + NBO x 2	311/12 years + NBO x 2	Blurry vision, brain fog, decreased concentration	397
55	13F	Viral encephalitis, seizure disorder	15 mos.	1.2/60 x 35 bid; 1.1/60 x 6 qod, 1.1/60 x 37 qd	33; 64	33; 23	Initial improvement then increase in aggression, violence, and loss of other gains; Increased seizure frequency, agitation, aggression,	40; 74

							decreased sleep, regression of gains	
56	25M	TBI, SDH, SAH	16 mos.	1.4/50 x 39 qd, 1.4/50 x 17; 1.15/45 x 18; 1.3 air/90 x 24, qd, 8.5y break, 1.15/45 x 1; 1.3/45 air x 7	56; 69; 99	17; 13; 1	Behavior regression with increased agitation, confusion, cognitive decline, elopement, slamming doors; increased agitation in chamber, rubbing head vigorously, yelling, disrupted sleep, loud voice, vigorous skin rubbing, bouncing legs; agitation in chamber, grimacing, baring teeth, rubbing head	65; 76; 91
57	2.5M	Post-drowning, cardiac arrest	3 mos.	Intermittent NBO 2L O2 nasal cannula/45min bid x 8, HBA: 1.3/45 x 20 qd, HBOT: 1.15/45 x7 qd; HBA 1.3/45 x2; HBA 1.15/45 x 1; HBA 1.15/50 x 12	NBO + 27; 29; 30; 32	NBO +27; 2; 1; 2	Diaphoresis, increased arching with myoclonus by 7th O_2 treatment, agitation, unable to sleep for 48 hrs; increased neurostorming, no sleep x 72h; neurostorming, spasms, opisthotonos;	12; 12.4; 12.6; 13

							agitation, inability to sleep, tachycardia to 170-180s, prolonged wakefulness	
58	22F	Lyme, Auto-Immune Encephalopathy/ Meningitis, Herpes Encephalitis (HE), Electrographic seizure disorder	2.5 mos. post HE	1.15/45 x 40; 15 mos. later: 1.15/45 x 1	41	1	Lip twitching, jaw spasm/trismus, gross arm tremor 10 mins. into treatment	35
59	83F	Stroke, persistent dizziness and short-term memory loss	11y	1.5/60 x 21, 1.3(95% O2)/60 x 229 qd 10 liters/min non-rebreather mask	156	135	Increased agitation, irritability, paranoia, combativeness, and anger	198

Table 5. Single case of Withdrawal Syndrome. **BOLD:** case treated at outside facility. Subject # corresponds to subject #'s in Supplementary File S2. HBOT schedule: x ATA/y = pressure level in atmospheres absolute/minutes of total treatment time unless specifically designated. qd=once/day, bid=twice/day.

Sub-ject	Age/Sex	Diagnosis	Time from injury to HBOT	HBOT Schedule	Num-ber of HBOTs at Symp-	# of consec-utive HBOTs at Symp-	Signs/Symptoms of Ox Tox	Cumu-lative HBOT dose

					tom Onset	tom Onset		(ATA-hrs.)
**60**	Young child, F	Dystonia	Years	1.75/60, bid, x 40; 1.75/60, bid, x 20; 1.75/60, bid, 7d/wk., x 90	150	90	Marked improvement in dystonia during each block of treatment, complete regression over 1 month break between blocks. By end of 3rd block of HBOT: "Very energetic, active, healthy, and relaxed with improvement in dystonia again." After 2 week break reversal, extreme rigidity, twisted, "her legs are turning, her pelvis is deviated, her left hip….she in not eating well, constant total body involuntary movements, teeth-grinding."	263
